# Long-term survival in patients with brain metastases—clinical characterization of a rare scenario

**DOI:** 10.1007/s00066-023-02123-4

**Published:** 2023-08-30

**Authors:** M. Hügel, J. Stöhr, T. Kuhnt, F. Nägler, K. Papsdorf, S. Klagges, P. Hambsch, E. Güresir, N. H. Nicolay, C. Seidel

**Affiliations:** 1 Department of Radiation Oncology, University of Leipzig Medical Center, Leipzig, Germany; 2 Clinical Cancer Registry, Leipzig, Germany; 3 Department of Neurosurgery, University of Leipzig Medical Center, Leipzig, Germany

**Keywords:** Stereotactic radiotherapy, Whole brain radiotherapy, Systemic therapy, ds-GPA, Frailty

## Abstract

**Purpose:**

This study aimed to assess clinical, treatment, and prognostic features in patients with brain metastases (BM) from solid tumors achieving long-term survival (LTS). Further, the accuracy of diagnosis-specific Graded Prognostic Assessment scores (ds-GPA) to predict LTS was evaluated.

**Methods:**

Patients admitted for radiotherapy of BM between 2010 and 2020 at a large tertiary cancer center with survival of at least 3 years from diagnosis of BM were included. Patient, tumor, treatment characteristics and ds-GPA were compiled retrospectively.

**Results:**

From a total of 1248 patients with BM, 61 (4.9%) survived ≥ 3 years. In 40 patients, detailed patient charts were available. Among LTS patients, median survival time from diagnosis of BM was 51.5 months. Most frequent primary tumors were lung cancer (45%), melanoma (20%), and breast cancer (17.5%). At the time of diagnosis of BM, 11/40 patients (27.5%) had oligometastatic disease. Estimated mean survival time based on ds-GPA was 19.7 months (in 8 cases estimated survival < 12 months). Resection followed by focal or whole-brain radiotherapy (WBRT) was often applied (60%), followed by primary stereotactic radiotherapy (SRT) (20%) or WBRT (20%). 80% of patients received systemic treatment, appearing particularly active in specifically altered non-small lung cancer (NSCLC), melanoma, and HER2-positive breast cancer. Karnofsky performance score (KPS) and the presence of oligometastatic disease at BM diagnosis were persisting prognostic factors in LTS patients.

**Conclusion:**

In this monocentric setting reflecting daily pattern of care, LTS with BM is heterogeneous and difficult to predict. Effective local treatment and modern systemic therapies often appear crucial for LTS. The impact of concomitant diseases and frailty is not clear.

**Supplementary Information:**

The online version of this article (10.1007/s00066-023-02123-4) contains supplementary material, which is available to authorized users.

## Introduction

Brain metastases (BM) are frequent complications in patients with advanced solid tumors [1–3]. The propensity for metastatic spread to the brain varies widely among tumor types, being highest among patients with lung cancer [3–5]. With up to 30% of all cancer patients affected [6–8], BM are the most common brain malignancies in adults [2, 4, 9] and their diagnosis often marks a turning point in the therapeutic approach and prognosis [3].

The frequency of patients diagnosed with BM has increased in recent decades and is expected to rise further [4, 10]. Apart from an improved prognosis of primary tumors, better imaging and the increased availability of magnetic resonance imaging (MRI) facilitates detection of brain lesions [8, 9, 11–13].

Regarding prognosis, median survival for patients with BM is still poor despite improved treatment options for primary tumors over the past years [2–4, 14, 15].

Factors determining survival time after a primary diagnosis of BM are known to a very limited extend. In particular, long-term survival (LTS) after diagnosis of BM is poorly characterized.

Prognostic scores, such as the disease-specific Graded Prognostic Assessment (ds-GPA) score, are used as an aid to predict survival time [16]. These scores mainly utilize parameters like the Karnofsky performance score (KPS), age, number of BM, and tumor subtype. It is unclear whether existing prognostic scores can predict LTS satisfactorily.

Little is known about the impact of other conceivable factors like concurrent diseases and treatment effects in long-term surviving patients. Our aim was to characterize clinical characteristics, treatment patterns, and the value of prognostic factors in detail in long-term surviving patients with BM and to analyze whether LTS is adequately reflected in ds-GPA sores.

## Materials and methods

This study was performed according to the principles of the Declaration of Helsinki. Approval was granted by the ethics committee of the Medical Faculty of the University of Leipzig (date 03.08.2021, no. 332/21-ek). All patients consented to anonymized scientific use of their clinical data. Patients who were treated with brain-directed radiotherapy at the Department of Radiotherapy at the University of Leipzig Medical Center between 2010 and 2020 were identified from clinical records. All patients (age ≥ 18 years) diagnosed with BM from a solid primary and with a survival time of ≥ 3 years from diagnosis of BM to date of death or last follow-up were eligible for the study. Survival time was defined as time from diagnosis of BM to death or loss to follow-up and was tabulated according to the Kaplan–Meier method. Differences in survival time were analyzed using a two-sample *t*-test. Patient data were analyzed from existing patient charts. Various variables including age (at primary cancer and BM diagnosis), sex, primary tumor, and histological subtype, KPS, activity of systemic disease immediately prior to diagnosis of BM, ds-GPA score, time interval between diagnosis of primary and BM, number of BM, treatment modalities, and concurrent diseases were evaluated. Score-based estimated survival time was calculated through ds-GPA score; ds-GPA was determined according to Sperduto et al. [16, 17] for NSCLC adeno- and non-adenocarcinoma, breast cancer, melanoma, renal cell carcinoma, and gastrointestinal (GI) cancer. In other tumor entities, the ds-GPA score was not applicable. Required items used for ds-GPA are displayed in Supplement 1.

Survival was analyzed using the Kaplan–Meier method (α = 0.05). For comparative analysis, univariate and multivariate Cox regression and log-rank tests were used. Data analysis was performed with Microsoft Excel 2016 (Microsoft corporation, Redmond, Washington, USA) and GraphPad Prism version 9.3.1 (350; GraphPad Software Inc., La Jolla, CA, USA).

## Results

### Patients without LTS

A total of 1248 patients were treated in the described period. 1187 of them did not reach LTS. Among them, 659 (55.5%) were male and 528 (44.5%) were female. Median age at diagnosis of the primary tumor was 62.09 years (mean 60.97, range 7–90). Median age at diagnosis of BM was 63.64 years (mean 62.78, range 18–90).

The distribution of primary tumors was as follows: lung cancer (NSCLC and SCLC): 609 patients (51.3%), breast cancer: 122 patients (10.3%), melanoma: 164 patients (13.8%), renal cancer: 118 (9.9%), colorectal cancer: 40 patients (3.4%), other: 134 (11.3%).

Median survival time from diagnosis of BM was 5 months (mean 7.8, range 12–115). Mean follow-up of the entire cohort was 7.0 years.

### Survival and patient characteristics in LTS

**Fig. 1:**
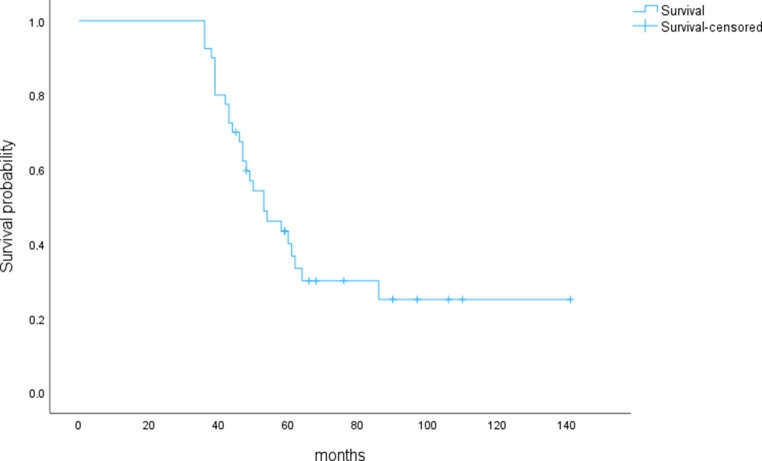
Survival in months after diagnosis of brain metastases

Of 1248 patients, 61 patients (4.9%) survived 3 years or longer from the time of diagnosis of BM. In 40 patients with LTS, detailed charts were available, among whom median (mean, range) survival time from diagnosis of BM was 51.5 months (58.4 months, 36–141 months). 24 patients were male (60%) and 16 female (40%). Data are summarized in Fig. 1 and Table 1.

**Table 1:** Patient characteristics

*Age at diagnosis of BM (years); median (range)*	59 (39–83)
*Duration from diagnosis of primary tumor to BM (months); median (range)*	9.5 (0–164)
*Gender; male (%), female (%)*	24 (60), 16 (40)
*KPS at diagnosis of BM; median (range)*	90 (60–100)
*Number of BM at diagnosis of BM; n (% of patients)*	
1	20 (50)
2–3	10 (25)
> 4	10 (25)
*Systemic tumor progression at diagnosis of BM; n (% of patients)*	
Yes (including synchronous BM)	31 (77.5)
No	9 (22.5)
*Synchronous BM; n (% of patients)*	17 (42.5)
*Metachronous BM; n (% of patients)*	23 (57.5)
*Survival time from diagnosis of BM (months); median (range)*	51.5 (36–141)
*Concurrent diseases; n (% of patients)*	
Smoking	18 (45)
Arterial hypertension	19 (47.5)
Hypercholesteremia	4 (10)
Peripheral arterial occlusive disease	2 (5)
Diabetes mellitus	3 (7.5)
Other cardiovascular diseases	9 (22.5)
COPD	6 (15)
History of other tumor disease	10 (25)

*BM *brain metastases, *COPD *chronic obstructive pulmonary disease

Mean age at initial diagnosis of primary tumor was 57.7 years. Age of patients at diagnosis of BM ranged from 39 to 83 years, with median and mean age of 59 and 59.7 years, respectively.

Compared to non-LTS patients, mean age in LTS patients was significantly lower (*p* = 0.043, two-sample *t*-test).

Duration from diagnosis of primary tumor to presentation with BM ranged from 0 to 164 months, with a median time of 9.5 months (mean 24.8 months) and varied according to tumor entity.

KPS at initial diagnosis of BM was 60% in one case, 70% in 5 cases, 80% in 9 cases, 80–90% in one case, 90% in 15 cases, and 100% in 9 cases (median 90%).

Overall, half of the patients were diagnosed with a single BM (20/40; 50%), whereas 2–3 BM were found in 25% (10/40) and ≥ 4 BM in 25% (10/40) of cases.

Immediately prior to diagnosis of BM, 77.5% (31/40) of patients had progressive systemic disease (unstable), as progression of primary or newly diagnosed/progressive metastases outside the brain, whereas 9 patients (22.5%) showed stable disease. Patients with synchronous BM were considered as unstable.

Whereas 42.5% (17/40) of patients were diagnosed with BM synchronous to the primary tumor, 57.5% (23/40) showed metachronous BM.

At the time of diagnosis of BM, 11/40 patients (27.5%) had oligometastatic disease with maximally four metastases.

Concurrent diseases were arterial hypertension (47.5%), hypercholesteremia (10%), peripheral arterial occlusive disease (5%), chronic obstructive lung disease (COPD; 15%), other cardiovascular diseases (22.5%), and diabetes mellitus (7.5%); 45% of patients were smokers (Table 1).

### Histologic and molecular features of primary tumors and BM

The most common primary tumor was lung cancer in 18/40 patients (45%), followed by melanoma (8/40; 20%), breast cancer (7/40; 17.5%), renal cell carcinoma (5/40; 12.5%), colorectal cancer (1/40; 2.5%), and bladder cancer (1/40; 2.5%).

**Table 2:** Tumor subtypes

Primary tumor	Subtype	Results (%)
NSCLC (*n* = 15)	*Adenocarcinoma*	13 (86.7)
	*EGFR* and/or *ALK* mutation	4 (30.8)
	*EGFR*/*ALK* wildtype	7 (53.8)
	*EGFR*/*ALK* status unknown	2 (15.4)
	*Squamous cell carcinoma*	2 (13.3)
Melanoma (*n* = 8)	*BRAF-V600E *mutation	4 (50)
	*BRAF-V600E *wildtype	4 (50)
Breast cancer (*n* = 7)	*Luminal A*	1 (14.3)
	*Luminal B (HER2-positive)*	2 (28.6)
	*HER2-positive*	3 (42.9)
	*Triple-negative*	1 (14.3)

*NSCLC* non-small cell lung cancer, *EGFR* epidermal growth factor receptor, *ALK* anaplastic lymphoma kinase, *HER2* human epidermal growth factor receptor 2

Tumor subtypes among patients with lung cancer were non-small cell lung cancer (NSCLC) in 15/18 cases (83.3%) and small cell lung cancer (SCLC), sarcomatoid carcinoma, and adenocarcinoma (of the lung) not further specified in one case each (5.6%). The histologies/subtypes in breast cancer/NSCLC/melanoma are displayed in Table 2*.*

The distribution of the tumor types lung cancer, melanoma, breast cancer, renal cell carcinoma, and colorectal cancer was not different in LTS patients compared to non-LTS (*p* = 0.557, chi-square).

### Tumor-specific survival and value of initial prognostic parameters among LTS patients

Tumor-specific mean time to BM occurrence was 6.6 months for lung cancer (of those, 12/18 with synchronous BM), 31.5 months for melanoma (0/8 with synchronous BM), 45.1 months for breast cancer (3/7 with synchronous BM), 30.4 months for renal cell carcinoma (2/5 with synchronous BM), 25 months for colon cancer, and 127 months for bladder cancer.

At termination of data acquisition, 13 patients (32.5%) were alive.

Tumor-specific mean survival time from diagnosis of BM was 57.3 months among patients with lung cancer, 66.1 months among patients with melanoma, 62.1 months among patients with breast cancer, and 49.4 months among patients with renal cell carcinoma.

**Fig. 2:**
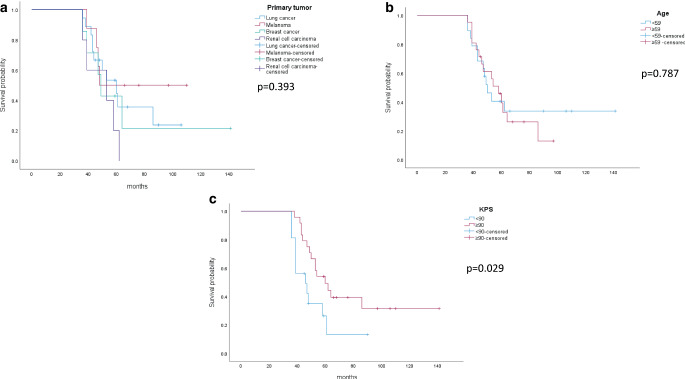
Survival among long-term survival patients depending on primary tumor (**a**), patient age (**b**), and KPS (**c**)

In univariate Cox regression analysis, survival was not significantly different between patients with different tumor entities (Fig. 2a, *p* = 0.398, log-rank). Age at presentation of BM (above/below median of 59 years) was not associated with survival (Fig. 2b, *p* = 0.783) while worse KPS (< 90) appeared to be associated with shorter survival (Fig. 2c, *p* = 0.029).

**Fig. 3:**
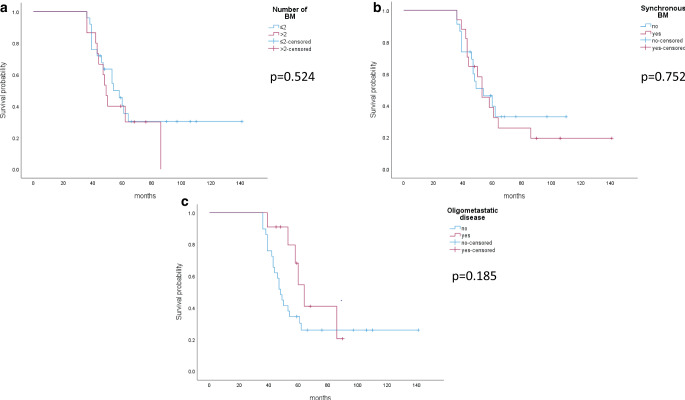
Survival among long-term survival patients depending on number (**a**) and timing of brain metastasis (**b**) and oligometastasis (**c**)

Regarding relevance of number (≤ 2 vs. > 2, *p* = 0.524) and timing of BM (synchronous vs. metachronous, *p* = 0.752) and oligometastatic disease (≤ 4 metastases vs. polymetastatic disease, *p* = 0.185), no significant effects were observed (Fig. 3a, b, and c).

In multivariate Cox regression analysis with the mentioned covariates (tumor type, age, KPS, number and timing of BM, presence of oligometastatic disease) only KPS (*p* = 0.004) and presence of oligometastatic disease (*p* = 0.036) were significantly associated with survival.

### Predicted survival with disease-specific Graded Prognostic Assessment

Expected mean survival time was assessable with ds-GPA for 36/40 patients and was 19.7 months (range 4–46 months). For 8/36 cases (22.2%), the estimated survival time was shorter than 12 months. A survival of 3 or more years was correctly predicted for 2/13 patients (15.4%) with NSCLC adenocarcinoma of the lung and for 1/7 patients (14.3%) with breast cancer. In the majority of patients, the ds-GPA score did not predict survival time correctly. Exemplary, short-term survival of below 1 year was predicted in 1/13 patients (7.7%) with NSCLC adenocarcinoma of the lung, 1/2 patients (50%) with NSCLC squamous cell carcinoma of the lung, 4/8 patients (50%) with melanoma, 1/5 patients (20%) with renal cell carcinoma, and 1/1 patient (100%) with colon cancer. A detailed summary of all ds-GPA scores and predicted survival times is displayed in Table 3.

**Table 3:** Distribution of retrospectively assessed disease-specific Graded Prognostic Assessment (ds-GPA) scores among patients per tumor entity

Primary tumor	Ds-GPA score	Expected survival time (months)	Number of patient (%)
NSCLC adenocarcinoma (*n* = 13)	0.5	7	1 (7.7)
	1.5	13	2 (15.4)
	2.5	25	3 (23.1)
	3	25	5 (38.5)
	4	46	2 (15.4)
NSCLC squamous cell carcinoma (*n* = 2)	2	10	1 (50)
	2.5	13	1 (50)
Melanoma (*n* = 8)	0.5	5	1 (12.5)
	2	8	3 (37.5)
	2.5	16	2 (25)
	3	16	1 (12.5)
	4	34	1 (12.5)
Breast cancer (*n* = 7)	2	13	2 (28.6)
	2.5	24	2 (28.6)
	3	24	2 (28.6)
	3.5	36	1 (14.3)
Renal cell carcinoma (*n* = 5)	1	4	1 (20)
	2	12	2 (40)
	3	17	1 (20)
	3.5	35	1 (20)
Gastrointestinal cancer (colon cancer; *n* = 1)	2.5	11	1 (100)

*NSCLC* non-small cell lung cancer, *ds-GPA* disease-specific Graded Prognostic Assessment

### Local treatment/radiotherapy

All included patients received radiation of the BMs. As initial therapy, resection followed by focal radiotherapy of the metastasis bed (18 patients, 45%) or by WBRT (6 patients, 15%) was the chosen option in the majority of cases (24/40, 60%). Less frequent was sole SRS of metastases (8/40, 20%) and WBRT (8/40, 20%). For details see (Table 4).

**Table 4:** Details of initial intracranial treatment and number of brain metastases

Intracranial treatment	Number of patients (%)	Number of brain metastases (%)		
		1	2–3	≥ 4
*Combined treatment*				
Surgery with radiotherapy of metastasis bed (3DCRT)	11 (27.5)	9 (81.8)	2 (18.2)	0 (0)
Surgery with radiotherapy of metastasis bed (IMRT)	1 (2.5)	1 (100)	0 (0)	0 (0)
Surgery with radiotherapy of metastasis bed (SRT)	4 (10)	2 (50)	2 (50)	0 (0)
Surgery with WBRT	2 (5)	1 (50)	0 (0)	1 (50)
Surgery with WBRT and SRT boost	4 (10)	0 (0)	2 (50)	2 (50)
Surgery with radiotherapy of metastasis bed (3DCRT) and SRT of other lesions	2 (5)	0 (0)	1 (50)	1 (50)
*Radiotherapy only*				
SRT	8 (20)	6 (75)	1 (12.5)	1 (12.5)
WBRT	6 (15)	1 (16.7)	2 (33.3)	3 (50)
WBRT with SRT boost	2 (5)	0 (0)	0 (0)	2 (100)

*3DCRT* three-dimensional conformal radiotherapy, *IMRT* intensity-modulated radiotherapy, *SRT* stereotactic radiotherapy, *WBRT* whole-brain radiotherapy

Among patients who underwent resection followed by radiotherapy, those with only one lesion comprised 54.2% (13/24). 10/13 patients (77%) had a completely resected brain metastasis according to postoperative MRI. Among patients who underwent WBRT, the majority presented with ≥ 4 BM (5/8, 62.5%).

Among patients with SRS, 6/8 patients had a single BM, one patient had 3 BM, and one patient ≥ 4 BM.

Among patients receiving WBRT only, 5/8 patients had ≥ 4 BM, 2 patients had 2 BM, and one patient had a single BM.

During the later course of disease, 26/40 patients developed intracranial progression and 19/40 patients developed systemic progression.

The mean time to intracranial progression after radiotherapy of the brain was 25.8 months, while the mean time to systemic progression was 25.3 months.

Survival time among long-term survivors not operated for BM was not different between patients treated with SRS or WBRT (51.8 months vs. 49.6 months, *p* = 0.486).

### Systemic treatment

Of the 40 patients, 32 (80%) received systemic treatment, the majority (20/32; 62.5%) started after completion of radiotherapy of the brain. Patient-specific details of treatment (sequence and duration of systemic treatments) are displayed in Supplements 2 and 3.

The treatments of the three largest patient groups (adenocarcinoma of the lung, melanoma, HER2-pos breast cancer) are described here in more detail:

Of the 15 patients with NSCLC, 33.3% (5/15) received EGFR-targeting tyrosine kinase inhibitors (erlotinib, afatinib, osimertinib, lapatinib) at some time after diagnosis of BM. Median duration of this medication was 38 months.

A single patient with an *EML4-ALK* fusion received crizotinib for an (estimated) period of 31 months.

Of the 15 patients with NSCLC, 9 received mostly short-lasting conventional chemotherapies involving, e.g., cis-/carboplatin or docetaxel.

In melanoma, 5/8 patients (62.5%) received immune checkpoint inhibition (ICI), i.e., pembrolizumab or ipilimumab ± nivolumab as treatment at some time after diagnosis of BM. Median duration of this treatment was 9 months. Only one patient with a *BRAF-V600E *mutation received a specific inhibitor (vemurafenib) for 10 months.

In breast cancer, 60% (3/5) of patients with HER2-positive disease received anti-HER2 blockage (trastuzumab or lapatinib) after diagnosis of BM, with a median treatment duration of 54 months.

## Discussion

Long-term survival with BM was encountered in very few patients in our series. This observation is in agreement with other authors who have described survival of more than 3 years in 2–3.3% of patients [10, 18]. To define LTS as a survival time of more than 3 years is somewhat arbitrary but in line with the prior publications [10, 17]. The most frequent tumor entities among long-term survivors were NSCLC (adenocarcinoma), melanoma, and breast cancer. Independent of survival time, lung cancer, breast cancer, and melanoma are the most common sources of BM [4, 19–21]; this might largely reflect the frequency of patients with BM in the disease and not a proneness to LTS in these entities. In our cohort, the distribution of tumor types among LTS patients resembled that of non-LTS patients. Likewise, in another recent series, NSCLC, breast cancer, and melanoma were the most frequent primary tumors among patients with LTS [22]. In other studies, survival of 3 years after diagnosis of BM occurred in 2.6% (lung), 2% (breast), and 3.3% (melanoma) patients [10, 18].

Although mean age was significantly lower in LTS than in non-LTS patients, there was considerable heterogeneity regarding age, course of disease, number of metastases, and type of local and systemic treatments in LTS patients.

Retrospectively calculated ds-GPA scores appeared not to be able to predict the LTS of our patients with precision. The principal elements of the initial GPA score from 2008 were age, KPS, extracranial metastases, and the number of BM [23]. Later, more specific prognostic factors for the five most frequent primary tumor entities (non-small and small cell lung cancer, melanoma, breast cancer, renal cell carcinoma, and gastrointestinal cancer) were introduced in disease-specific GPA scores (Supplement 1) [24, 25]. However, as a score based on only few clinical items, it appears inherently more accurate for the large majority of patients with survival near to median survival and will lose accuracy at the upper and lower end of survival time. Hence, ds-GPA will likely allow prognostic assessment for the majority but not for all patients. In our series, LTS of ≥ 3 years does not appear to be well covered with ds-GPA. Apart from statistical reasons there might be several contributing factors to this. Firstly, therapeutic aspects like particular sensitivity to local or systemic treatment are not reflected in the score. Secondly, other pretreatment factors, e.g., accurately reflecting frailty [26], are missing in the score. However, relevant positive prognostic factors of ds-GPA like KPS (median 90%), a young age (median 59 years), and non-disseminated BM (below 4 BM: 75% of patients) were frequent among LTS patients [27]. For the clinical setting, cautious use of ds-GPA appears desirable.

Regarding the effect of local treatment, the majority (60%) of patients received resection prior to radiotherapy, which is common practice within the DEGRO Radiosurgery and Stereotactic Radiotherapy Working Group [28]. In clinical trials applying this approach, a median survival of 14 months is achieved [29], proving the efficacy of this treatment.

In our series, 35% of patients remained intracranially stable after radiotherapy and the mean time to intracranial progression was 25.8 months, i.e., particularly long compared to other studies [19]. Most likely this reflects particular sensitivity to local and/or systemic treatment active in the brain. Interestingly, also some patients with multiple BM after WBRT were among the long-term survivors. It is well known that LTS may occur after WBRT for multiple metastases, but this is very rare [18]. Remarkably, 8/40 patients (20%) reached LTS after local treatment without any further systemic treatment.

In recent years, several new treatment modalities in oncology have opened up new prognostic horizons. In our cohort, three larger subgroups (adenocarcinoma of the lung with specific mutations, melanoma, HER2-positive breast cancer) in which new treatments have been introduced were highly represented.

Overall, 40% of NSCLC patients received either a treatment with specific EGFR inhibitors (median treatment duration of 38 months) or crizotinib (median duration of 31 months) for an *EML4-ALK* fusion. It is well known that these treatments are highly active in BM, and PFS in recent trials with BM ranged from 8.6 to 16.5 months [30–35]. With this background it appears unsurprising that in LTS patients, targetable alterations are much more frequent than among non-selected patients with adenocarcinoma of the lung (EGFR: 41 vs. 11%; ALK: 8 vs. 4.8%) [22, 36]. However, the majority of NSCLC patients still showed a histology of adenocarcinoma without a specific alteration and reached LTS without specific systemic treatment.

In melanoma, 62.5% of patients received ICI as treatment at some time after the diagnosis of BM (median duration of treatment 9 months). Only one patient with a *BRAF-V600E* mutation received a specific inhibitor (vemurafenib) for 10 months. Several trials indicated high activity of ICI in BM, with around 30% of patients achieving durable responses to ICI [37], intracranial PFS of 59.5% after at 9 months [38], and 5‑year intracranial PFS of 14–52% (Rx naïve) [39]. Our results underline that ICI can contribute to LTS in melanoma with BM, which would otherwise be a rapidly fatal disease in most cases [40, 41].

In breast cancer, 60% (3/5) of patients with HER2-positive disease received trastuzumab after the diagnosis of BM, with a median treatment duration of 32 months. Survival under several cytotoxic treatments in this patient group was shorter, mainly in the range of several months.

HER2 targeting is a successful story in the treatment of breast cancer, with firstly trastuzumab and lapatinib being active in HER2-positive BM patients [42, 43]. More recently, other substances like tucatinib [44] or antibody–drug conjugates like trastuzumab deruxtecan [45–47] proved to be active in BM.

In summary, the effect of systemic treatment on LTS appears considerable and is currently not adequately reflected in prognostic scores.

The role of other concomitant diseases or conditions for prognosis in patients with BM is largely unclear. Among LTS patients in our cohort, arterial hypertension, other cardiovascular diseases, COPD, and smoking were quite frequent, while diabetes mellitus was rather rarely encountered (7.5%). In a recent study, the presence of diabetes mellitus was a significant predictor of poorer overall survival in patients treated for BM with SRS [48]. Similar results were achieved in a recent retrospective analysis of our group (unpublished) showing that diabetes mellitus might be an independent negative prognostic factor in patients with BM. It appears likely that parameters that better reflect multimorbidity or frailty like the Hurria [49] or G8 score [50] may aid in prognostic assessment in BM patients. Further, it would be interesting to validate temporal muscle thickness, which appears to be a relevant independent prognostic parameter in patients with BM, [51, 52] in LTS.

The goal of this work was to generate data in a cohort of patients with characteristics that present to radiotherapy departments on a daily basis and not in the setting of a controlled clinical trial. This approach lacks the accuracy of a controlled clinical trial but can potentially better reflect to what extent the successes of clinical trials and innovations can be translated into a substantial patient benefit in the uncontrolled setting of daily practice involving routine radiotherapy. Our analysis thus has the limitations of a monocentric retrospective series with unfortunate loss of 1/3 of the patient data, but reflects clinical routine and can be an orientation for the treating radiotherapist. We find it somewhat surprising that the results are indeed so heterogenous and that “paths” to LTS can be so different. Larger series and a comprehensive comparison of non-LTS and LTS patients are needed to validated this finding in tumor-specific subgroups. In the absence of high sensitivity of ds-GPA, it is currently very hard to predict LTS. To exaggerate a bit: it is not just the very “fit” patient with very few metastases receiving stereotactic radiotherapy and a particular systemic treatment who can survive for longer than 3 years.

## Conclusion

Long-term survival in patients with BM is rare and difficult to predict with current scores. LTS patients are heterogeneous in their clinical features including number of metastases or age. Adequate local treatment appears important in many patients, as well as active systemic treatments. KPS at presentation of BM and oligometastatic disease keep their prognostic value in patients with LTS. The role of other scores encompassing concomitant diseases or frailty in BM remains to be evaluated in the future.

### Supplementary Information

Supplement 1 Items assessed for ds-GPA, score calculation, and estimated survival

Supplement 2 Overview of systemic treatments per patient

Supplement 3 Patient-specific course of disease and treatment
