# Supplementary material for: Long-term survival in patients with brain metastases—clinical characterization of a rare scenario
Source: Strahlenther Onkol. 2023 Aug 30;200(4):335–45. doi: 10.1007/s00066-023-02123-4 (PMC10965568; doi:10.1007/s00066-023-02123-4)
Supplement: Supplementary file 1 — Supplement 1 Items assessed for ds-GPA, score calculation, and estimated survival [file 66_2023_2123_MOESM1_ESM.docx]

**Supplement 1 Items assessed for ds GPA, score calculation and estimated survival**

| Prognostic factor by cancer type | GPA | | | | |  |
| --- | --- | --- | --- | --- | --- | --- |
|  | **0** | **0.5** | **1** | **1.5** | **2** | Sum and estimated survival in months |
| NSCLC adenocarcinoma |  | | | | | 0-1 = 7  1.5-2.0 = 13  2.5-3.0 = 25  3.5-4.0 = 46 |
| KPS | ≤70 | 80 | 90-100 |  |  |  |
| Age (years) | ≥70 | <70 |  |  |  |  |
| No. of BM | ≥5 | 1-4 |  |  |  |  |
| ECM | Present |  | Absent |  |  |  |
| EGFR and ALK | Both negative or unknown |  | EGFR or ALK positive |  |  |  |
| NSCLC nonadeno-carcinoma |  | | | | | 0-1 = 5  1.5-2.0 = 10  2.5-3.0 = 13 |
| KPS | ≤70 | 80 | 90-100 |  |  |  |
| Age (years) | ≥70 | <70 |  |  |  |  |
| No. of BM | ≥5 | 1-4 |  |  |  |  |
| ECM | Present |  | Absent |  |  |  |
| Melanoma |  | | | | | 0-1 = 5  1.5-2.0 = 8  2.5-3.0 = 16  3.5-4 = 34 |
| KPS | ≤70 | 80 | 90-100 |  |  |  |
| Age (years) | ≥70 | <70 |  |  |  |  |
| No. of BM | ≥5 | 2-4 | 1 |  |  |  |
| ECM | Present |  | Absent |  |  |  |
| BRAF | Negative or unknown | Positive |  |  |  |  |
| Breast cancer |  | | | | | 0-1= 6  1.5-2.0 = 13  2.5-3.0 = 24  3.5-4.0= 36 |
| KPS | ≤60 | 70-80 | 90-100 |  |  |  |
| Age (years) | ≥60 | <60 |  |  |  |  |
| No. of BM | ≥2 | 1 |  |  |  |  |
| ECM | Present | Absent |  |  |  |  |
| Subtype | Basal | Luminal A |  | HER2 or Luminal B |  |  |
| Renal cell carcinoma |  | | | | | 0-1 = 4  1.5-2.0 = 12  2.5-3.0 = 17  3.5-4.0 = 35 |
| KPS | ≤70 |  | 80 |  | 90-100 |  |
| No. of BM | ≥5 | 1-4 |  |  |  |  |
| ECM | Present | Absent |  |  |  |  |
| Hgb | <11.1 | 11.1-12.5 or unknown | > 12.5 |  |  |  |
| GI cancers |  | | | | | 0-1 = 3  1,5-2.0 = 7  2.5-3 = 11  3.5-4 = 17 |
| KPS | ≤70 |  | 80 |  | 90-100 |  |
| Age (years) | ≥60 | <60 |  |  |  |  |
| No. of BM | ≥4 | 2-3 | 1 |  |  |  |
| ECM | Present | Absent |  |  |  |  |

Abbreviations: BM, brain metastases; ECM, extracranial metastases; GPA, Graded Prognostic Assessment; Hgb, hemoglobin; KPS, Karnofsky performance status; MS, median survival; NA, not available; NSCLC, non–small-cell lung cancer.

From: Sperduto PW, Mesko S., Li J, et al. Survival in patients with brain metastases: Summary report on the updated diagnosis-specific graded prognostic assessment and definition of the eligibility quotient. J Clin Oncol 2020

**Supplement to article:**

**Long-term survival in patients with brain metastases – clinical characterization of a rare scenario**
