# Supplementary material for: Long-term survival in patients with brain metastases—clinical characterization of a rare scenario
Source: Strahlenther Onkol. 2023 Aug 30;200(4):335–45. doi: 10.1007/s00066-023-02123-4 (PMC10965568; doi:10.1007/s00066-023-02123-4)
Supplement: Supplementary file 2 — Supplement 2 Overview of systemic treatments per patient [file 66_2023_2123_MOESM2_ESM.docx]

**Supplement 2 Overview of Systemic Treatments per patient**

| **Patient** | **Histology** | **Subtype, driver mutation** | **Systemic treatment (group)** | **Systemic treatment (specific)** | **Duration** | **In months (*estimated)** |
| --- | --- | --- | --- | --- | --- | --- |
|  |  |  | **(Effect on brain metastases)** |  |  |  |
|  |  |  |  |  |  |  |
| V | NSCLC | AdenoCa, EGFR alteration | conventional chemotherapy | Carboplatin/Paclitaxel | starting 03/2011 (4 cycles) | 4* |
|  |  |  | anti-VEGF | Bevacizumab | 03/2011-05/2011 (2 cycles) | 2 |
|  |  |  | TKI | Erlotinib | 07/2011-approx. 27.03.2018 | 80* |
| BA | NSCLC | AdenoCa, EGFR alteration | conventional chemotherapy | Cisplatin/Pemetrexed | until 08/2015 (4 cycles) | 4* |
|  |  |  | TKI | Gefitinib | 01.04.2015-07/2015 | 3 |
|  |  |  | TKI | Afatinib | 08/2015-06/2018; unknown | ≥34* |
|  |  |  | TKI | Osimertinib | 07/2018-at least 16.03.2022 | ≥44* |
| BC | NSCLC | AdenoCa, EGFR alteration | conventional chemotherapy | Carboplatin/Pemetrexed | starting approx.03/2018 | unknown |
|  |  |  | TKI | Erlotinib | 12/2013-at least 08/2014 | ≥8* |
|  |  |  | TKI | Afatinib | 07/2015-01/2016; starting 14.09.2018 | ≥6* |
|  |  |  | TKI | Osimertinib | 03/2016-03/2018 | 24 |
| AQ | NSCLC | AdenoCa, EML-4-ALK alteration | conventional chemotherapy | Carboplatin/Pemetrexed | 09/2014 -11/2014 | 2 |
|  |  |  | TKI | Crizotinib | starting 11/2014 | 31* |
|  |  |  | TKI | Alectinib | starting 06/2017 | 26* |
|  |  |  | TKI | Lorlatinib | starting 08/2019-approx. 18.04.2020 | 8* |
| AB | NSCLC | AdenoCa, no known mutation | conventional chemotherapy | Cisplatin | 18.12.2013-03.04.2014 (6 cycles) | 3 |
|  |  |  | conventional chemotherapy | Pemetrexed | 18.12.2013-03.04.2014; until 01.09.2014 (12 cycles) | 8* |
|  |  |  | conventional chemotherapy | Docetaxel | approx. 01/2015-03/2015 | 2* |
|  |  |  | conventional chemotherapy | Paclitaxel | starting 02/2017 (at least 2 cycles) | ≥2* |
|  |  |  | anti-PD-L1 | Nivolumab | starting 02/2016 | 1* |
|  |  |  | anti-VEGF | Bevacizumab | starting 03/2016 | unknown |
|  |  |  | TKI | Nintedanib | approx. 01/2015-03/2015 | 2* |
|  |  |  | TKI | Erlotinib | starting 04/2015 | 12* |
|  |  |  | TKI | Osimertinib | starting after 03/2016-approx. 30.07.2017 | 15* |
| AN | NSCLC | AdenoCa, no mutation | conventional chemotherapy | Cispatin/Pemetrexed | 10/2015-03/2016 (6 cycles) | 5 |
| AS | NSCLC | AdenoCa, no mutation | conventional chemotherapy | Carboplatin/Pemetrexed | 03/2016-06/2016 | 3 |
|  |  |  | conventional chemotherapy | Docetaxel | 05.-24.08.2016 | 1 |
|  |  |  | anti-VEGF | Bevacizumab | 03/2016-06/2016 | 3 |
| AW | NSCLC | AdenoCa, no mutation | conventional chemotherapy | Cisplatin/Vinorelbine | 17.06.2014-28.07.2014 (2 cycles) | 1 |
|  |  |  | conventional chemotherapy | Docetaxel | 08/2016-12/2016 (6 cycles) | 4 |
|  |  |  | TKI | Nintedanib | 08/2016-12/2016 (6 cycles) | 4 |
| AY | NSCLC | AdenoCa, no mutation | conventional chemotherapy | Carboplatin | 16.12.2014-03.01.2015 | 1 |
| BB | NSCLC | AdenoCa, no mutation | conventional chemotherapy | Carboplatin/Paclitaxel | until 17.09.2012 (4 cycles) | 4* |
|  |  |  | anti-VEGF | Bevacizumab | until 25.02.2013 (9 cycles) | 9* |
| BD | NSCLC | AdenoCa, no mutation | conventional chemotherapy | Cisplatin/Pemetrexed | 04/2017-07/2017 (4 cycles) | 3 |
|  |  |  | conventional chemotherapy | Docetaxel | 07.07.2018-26.11.2018 (6 cycles) | 4 |
|  |  |  | anti-VEGF | Bevacizumab | 04/2017-02/2018 | 10 |
|  |  |  | anti-PD-L1 | Atezolizumab | 03/2018-04.05.2018 | 2 |
|  |  |  | TKI | Nintendanib | 07.07.2018-26.11.2018 (6 cycles); starting 01/2019-approx. 10.03.2020 | 18* |
| W | NSCLC | AdenoCa | conventional chemotherapy | Carboplatin/Vinorelbine | starting 25.07.2011, 4 cycles | 4* |
|  |  |  | conventional chemotherapy | Pemetrexed | 17 cycles, until 03/2013 | 17* |
|  |  |  | anti-VEGF | Bevacizumab | 17 cycles, until 03/2013 | 17* |
| AF | NSCLC | most likely Adeno-Ca, no mutation | conventional chemotherapy | Carboplatin/Paclitaxel | 07/2015-01/2016 (6 cycles) | 6 |
| AR | NSCLC | Squamous cell Carcinoma | conventional chemotherapy | Cisplatin/Docetaxel | 11/2016-09.12.2016 (2 cycles) | 1 |
| AC | SCLC |  | conventional chemotherapy | Carboplatin/Etoposide | starting 16.06.2014 (5 cycles) | 5* |
| X | Lung cancer (mixed subtype) | AdenoCa | conventional chemotherapy | Carboplatin/Paclitaxel | 08.09.2015 -01.10.2015 (2 cycles) | 1 |
|  |  |  | anti-VEGF | Bevacizumab | 08.09.2015-01.10.2015 (2 cycles) | 1 |
|  |  |  | TKI | Afatinib | starting 12/2016 | 20* |
|  |  |  | TKI | Lapatinib | starting 08/2018-approx. 29.04.2019 | 8* |
| AO | Lung cancer (sarcomatoid) |  | conventional chemotherapy | Carboplatin/Paclitaxel | 05/2014-08/2014 (4 cycles) | 3 |
| Z | Melanoma | BRAFV600E alteration | anti-PD-L1 | Pembrolizumab | >50 cycles (2.-50. cycle: 01/2016-01/2019) | ≥36* |
| AG | Melanoma | BRAFV600E alteration | anti-CTLA-4 | Ipilimumab | 02/2014-17.05.2014 (4 cycles) | 3 |
|  |  |  | anti-PD-L1 | Pembrolizumab | 15.06.2015 - at least 17.12.2015 (at least 7 cycles) | ≥6* |
|  |  |  | BRAF Inhibitor | Vemurafenib | 08/2014-06/2015 | 10 |
| AJ | Melanoma | No BRAF alteration | anti-PD-L1 | Nivolumab | 25.08.-06.10.2016 (3 cycles) | 1 |
|  |  |  | anti-CTLA-4 | Ipilimumab | 25.08.-06.10.2016 (3 cycles) | 1 |
| AA | Melanoma | No BRAF alteration | anti-PD-L1 | Pembrolizumab | 21.10.2014-20.09.2017 (48 cycles) | 35 |
|  |  |  | anti-CTLA-4 | Ipilimumab | 03/2014-05/2014 (4 cycles) | 2 |
| AL | Melanoma | No BRAF alteration | conventional chemotherapy | Dacarbazine | 04/2013-07/2013 | 3 |
|  |  |  | anti-CTLA-4 | Ipilimumab | 07/2013-11/2013 (4 cycles) | 4 |
| T | Melanoma | No BRAF alteration | conventional chemotherapy | Dacarbazine | 06/2011-02/2012; 14.03.2014-31.07.2014 (5 cycles) | 12 |
| AE | Breast cancer | Luminal A | conventional chemotherapy | Epirubicin/Cyclophosphamid/ Docetaxel | until 30.06.2004 | unknown |
|  |  |  | conventional chemotherapy | Capecitabine | 2016 | unknown |
|  |  |  | anti-HER2/neu | Lapatinib | 2016-at least 19.08.2021 | ≥67* |
| U | Breast cancer | Luminal B, Her2neu pos. | conventional chemotherapy | Paclitaxel | starting 08/2012 (at least 6 cycles planned) | ≥6* |
|  |  |  | anti-VEGF | Bevacizumab | unknown | unknown |
| AH | Breast cancer | Luminal B, Her2neu pos. | conventional chemotherapy | Epirubicin/Cyclophosphamid | until 03/2013 (4 cycles) | 4* |
|  |  |  | conventional chemotherapy | Paclitaxel | starting 04/2013 (8 cycles) | 8* |
|  |  |  | conventional chemotherapy | Emtansine | starting 03/2016; starting 03/2017 | unknown |
|  |  |  | conventional chemotherapy | Methotrexate | 02/2017 (3 cycles) | 3* |
|  |  |  | conventional chemotherapy | Cytarabine | 02/2017 (1 cycle) | 1* |
|  |  |  | anti-HER2/neu | Trastuzumab | 04/2013-02/2014; starting 03/2016; starting 03/2017 | 31* |
|  |  |  | anti-estrogen | Anastrozol | unknown | unknown |
|  |  |  | anti-HER2/neu | Lapatinib | 04/2014-03/2016 | 23 |
| AD | Breast cancer | HER2neu pos. | conventional chemotherapy | Paclitaxel | starting 04/2011 (12 cycles) | 12* |
|  |  |  | conventional chemotherapy | Navelbine | until 07/2013 | unknown |
|  |  |  | anti-HER2/neu | Trastuzumab | starting 07/2013-approx. 29.03.2016 (>12 cycles) | 32* |
| R | Breast cancer | Her2neu pos. | conventional chemotherapy | 5-Fluoruracil/Epirubicin/Cyclophosphamid/Docetaxel | 12.09.2005-03.01.2006 (3 cycles) | 3* |
|  |  |  | conventional chemotherapy | Capecitabine | 09/2011-04/2012 | 7 |
|  |  |  | conventional chemotherapy | Paclitaxel | 04/2010-08/2010; 6 cycles | 4 |
|  |  |  | conventional chemotherapy | Vinorelbine | 07/2013-10/2013; 05/2014-08/2014 | 6 |
|  |  |  | anti-HER2/neu | Trastuzumab | 1 year; at least 04/2010- 09/2011; 05/2012-01/2013; starting 07/2013 (at least until 09/2014) | ≥51* |
|  |  |  | anti-estrogen | Letrozol | 09/2010-09/2011 | 12 |
|  |  |  | anti-estrogen | Tamoxifen | 05/2012-01/2013 | 8 |
|  |  |  | anti-estrogen | Exemestan | 01/2013-07/2013 | 6 |
|  |  |  | anti-HER2/neu | Lapatinib | 09/2011-04/2012; 01/2013-07/2013; until 04/2014 | ≥13* |
| Y | Breast cancer | HER2neu pos. | conventional chemotherapy | Docetaxel/Doxorubicin/Cyclophosphamid | 07/2011-11/2011 | 4 |
|  |  |  | conventional chemotherapy | Capecitabine | 01.03.2016- at least 09/2016 | ≥6* |
|  |  |  | anti-HER2/neu | Trastuzumab | 01/2012-01/2013 | 12 |
|  |  |  | anti-VEGF | Bevacizumab | 01.03.2016- at least 09/2016 | ≥6* |
|  |  |  | anti-estrogen | Tamoxifen | 01/2013- at least 10/2015 | ≥33* |
| AU | Breast cancer | triple negative | conventional chemotherapy | Cispatin/Pemetrexed | 11.03.2010-04/2010 (3 cycles) | 1 |
|  |  |  | conventional chemotherapy | Docetaxel | 6 cycles | 6* |
|  |  |  | anti-VEGF | Bevacizumab | starting 01.03.2010 (6 cycles) | 6* |
| AK | Renal cell carcinoma | clear cell, PAX8/Vimentin/CD10 pos. | anti-PD-L1 | Nivolumab | starting 25.05.2016 | unknown |
|  |  |  | TKI | Sunitinib | starting 01/2009, 3 months | 3 |
|  |  |  | TKI | Pazopanib | starting 19.11.2015 (22.-30.12.2015 paused) | 3* |
|  |  |  | TKI | Everolimus | starting 18.03.2016 | 16* |
|  |  |  | TKI | Cabozantinib | starting 10.08.2017-approx. 02.07.2019 (15.02.2018-26.03.2018 paused) | 22* |
| AI | Renal cell carcinoma | clear cell/papillary | TKI | Sunitinib | starting 12/2013 | 25* |
|  |  |  | TKI | Everolimus | starting 2016-approx. 11.04.2018 | 27* |
| AT | Renal cell carcinoma | clear cell | anti-CD20 | Rituximab | 10/2015 1x | 1 |
| AZ | Renal cell carcinoma | clear cell | TKI | Pazopanib | 10.04.2014-04/2015 | 11 |
|  |  |  | TKI | Everolimus | starting 05/2015-approx. 02.05.2019 | 48* |
| AX | Renal cell carcinoma |  | TKI | Sunitinib | 13 cycles; unknown (planned) | ≥13* |
|  |  |  | TKI | Everolimus | unknown | unknown |
| Q | Bladder carcinoma |  | conventional chemotherapy | Gemcitabine/Cisplatin | until 06/2010 (6 cycles) | 6* |
|  |  |  | conventional chemotherapy | Vinflunine | until 01/2014 (12 cycles) | 12* |
| AM | Colon carcinoma |  | conventional chemotherapy | unknown | 01/2014 - 06/2014 | 5 |
|  |  |  | conventional chemotherapy | 5-Fluoruracil | starting 03.02.2016 | unknown |
|  |  |  | conventional chemotherapy | Irinotecan | at least 10/2019-07.02.2020 | ≥4* |
|  |  |  | conventional chemotherapy | Oxaliplatin | starting 03.02.2016; at least 10/2019-07.02.2020 | ≥4* |
|  |  |  | anti-EGFR | Cetuximab | 03.02.2016-at least 04/2018 | ≥25* |

**Supplement to article:**

**Long-term survival in patients with brain metastases – clinical characterization of a rare scenario**
