# Supplementary material for: Long-term survival in patients with brain metastases—clinical characterization of a rare scenario
Source: Strahlenther Onkol. 2023 Aug 30;200(4):335–45. doi: 10.1007/s00066-023-02123-4 (PMC10965568; doi:10.1007/s00066-023-02123-4)
Supplement: Supplementary file 3 — Supplement 3 Patient-specific course of disease and treatment [file 66_2023_2123_MOESM3_ESM.pptx]

## Slide 1
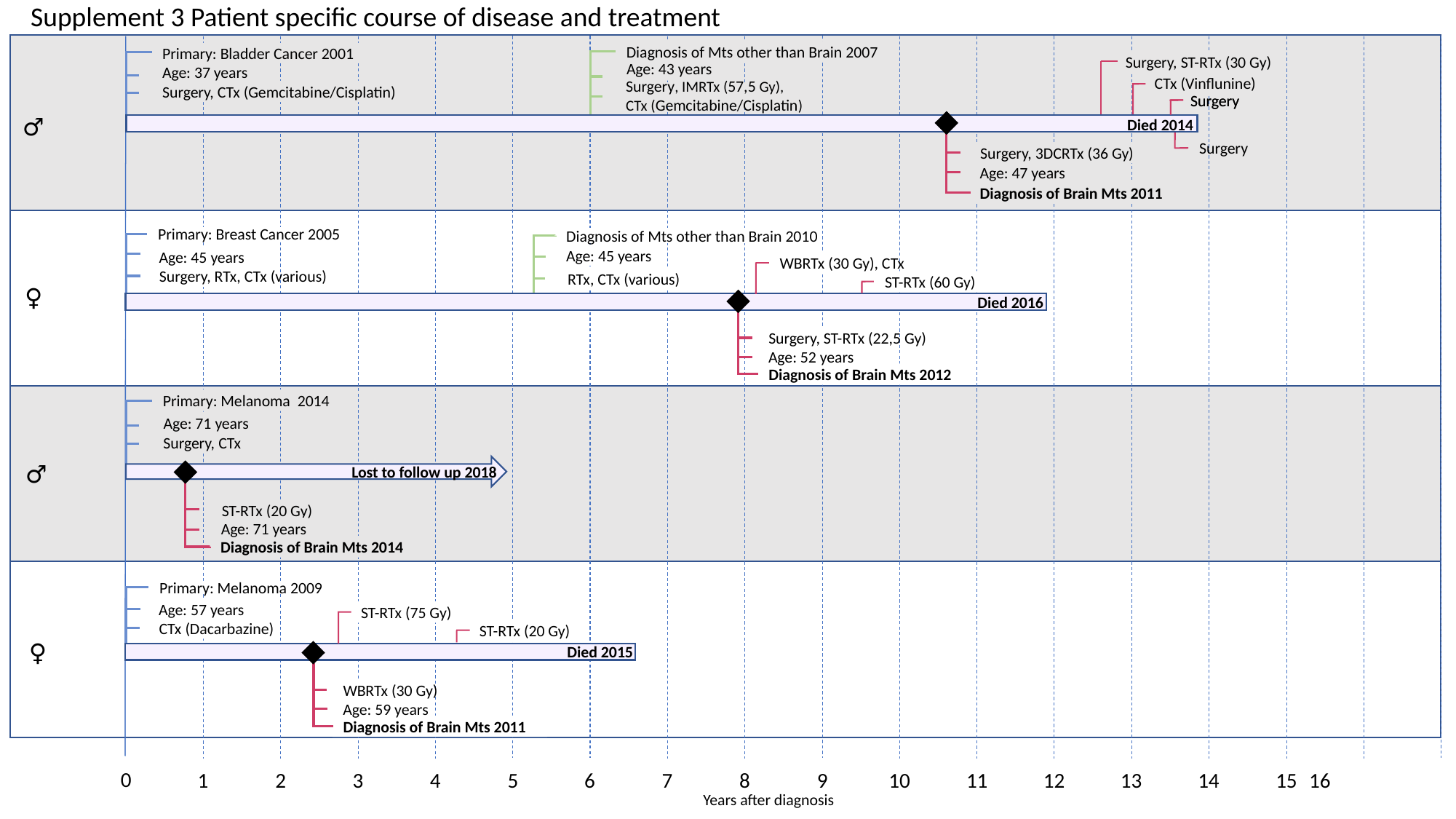

Supplement 3 Patient specific course of disease and treatment
Diagnosis of Mts other than Brain 2007
Primary: Bladder Cancer 2001
Surgery, ST-RTx (30 Gy)
Age: 43 years
Age: 37 years
CTx (Vinflunine)
Surgery, CTx (Gemcitabine/Cisplatin)
Surgery, IMRTx (57,5 Gy),
CTx (Gemcitabine/Cisplatin)
Surgery
Surgery
Died 2014
♂
Surgery
Surgery, 3DCRTx (36 Gy)
Age: 47 years
Diagnosis of Brain Mts 2011
Primary: Breast Cancer 2005
Diagnosis of Mts other than Brain 2010
Age: 45 years
Age: 45 years
WBRTx (30 Gy), CTx
Surgery, RTx, CTx (various)
RTx, CTx (various)
ST-RTx (60 Gy)
♀
Died 2016
Surgery, ST-RTx (22,5 Gy)
Age: 52 years
Diagnosis of Brain Mts 2012
Primary: Melanoma 2014
Age: 71 years
Surgery, CTx
Lost to follow up 2018
♂
ST-RTx (20 Gy)
Age: 71 years
Diagnosis of Brain Mts 2014
Primary: Melanoma 2009
Age: 57 years
ST-RTx (75 Gy)
CTx (Dacarbazine)
ST-RTx (20 Gy)
♀
Died 2015
WBRTx (30 Gy)
Age: 59 years
Diagnosis of Brain Mts 2011
0
1
5
2
15
10
4
13
8
11
6
7
9
12
14
3
16
Years after diagnosis

## Slide 2
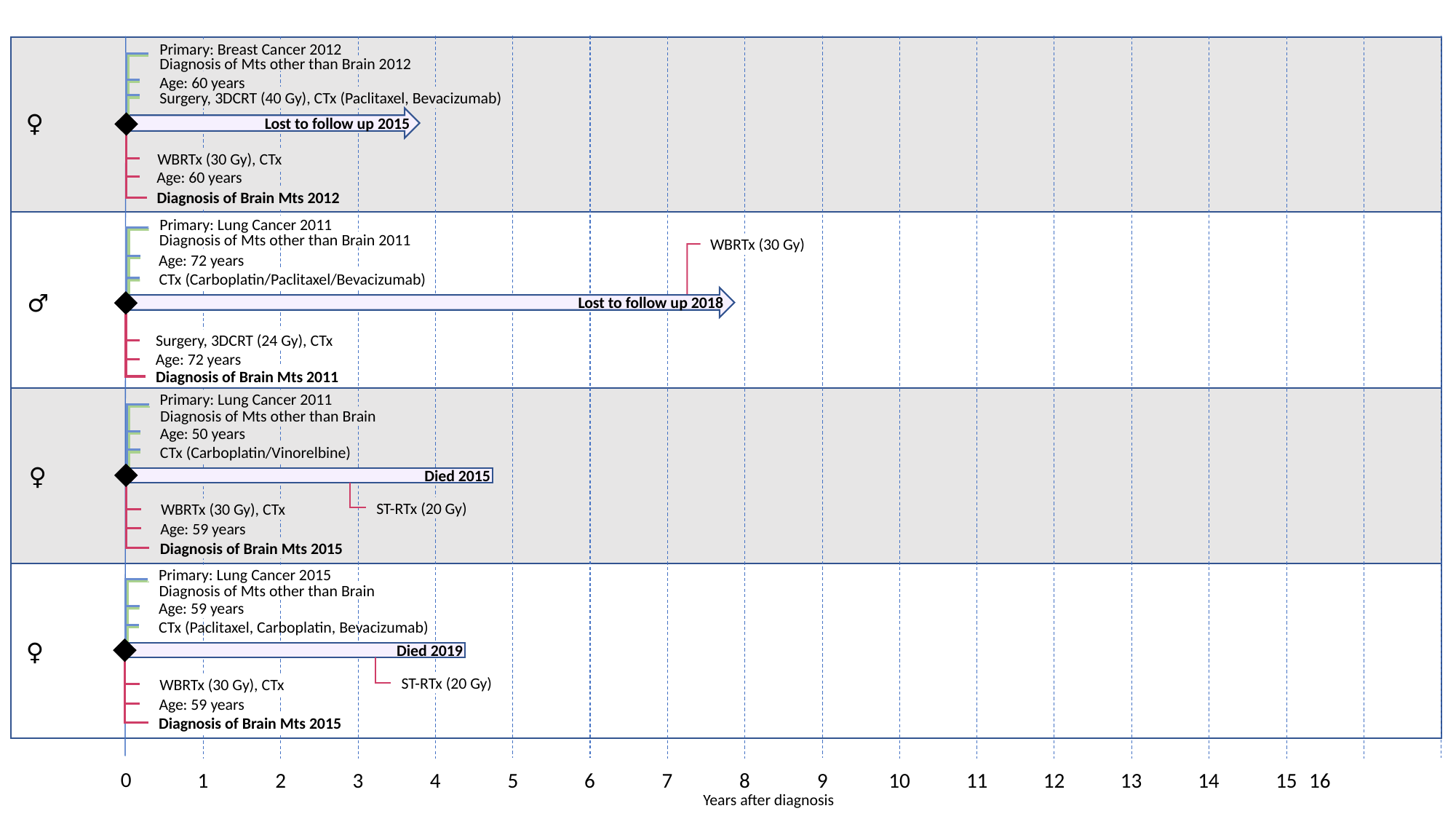

Primary: Breast Cancer 2012
Primary: Breast Cancer 2012
Diagnosis of Mts other than Brain 2012
Age: 60 years
Surgery, 3DCRT (40 Gy), CTx (Paclitaxel, Bevacizumab)
♀
Lost to follow up 2015
WBRTx (30 Gy), CTx
Age: 60 years
Diagnosis of Brain Mts 2012
Primary: Lung Cancer 2011
Diagnosis of Mts other than Brain 2011
WBRTx (30 Gy)
Age: 72 years
CTx (Carboplatin/Paclitaxel/Bevacizumab)
♂
Lost to follow up 2018
Surgery, 3DCRT (24 Gy), CTx
Age: 72 years
Diagnosis of Brain Mts 2011
Primary: Lung Cancer 2011
Diagnosis of Mts other than Brain
Age: 50 years
CTx (Carboplatin/Vinorelbine)
Died 2015
♀
ST-RTx (20 Gy)
WBRTx (30 Gy), CTx
Age: 59 years
Diagnosis of Brain Mts 2015
Primary: Lung Cancer 2015
Diagnosis of Mts other than Brain
Age: 59 years
CTx (Paclitaxel, Carboplatin, Bevacizumab)
Died 2019
♀
ST-RTx (20 Gy)
WBRTx (30 Gy), CTx
Age: 59 years
Diagnosis of Brain Mts 2015
0
1
5
2
15
10
4
13
8
11
6
7
9
12
14
3
16
Years after diagnosis

## Slide 3
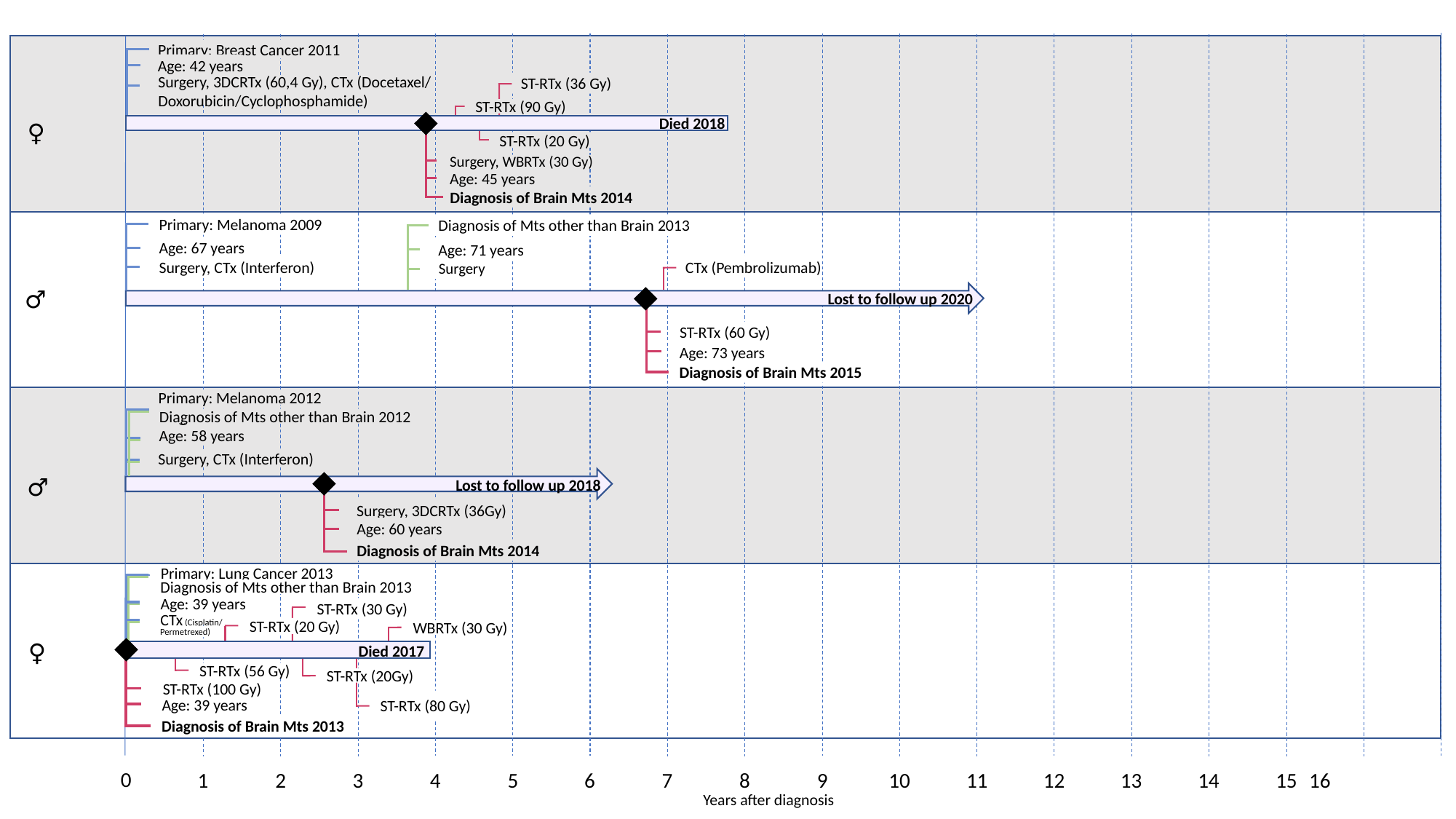

Primary: Breast Cancer 2011
Age: 42 years
ST-RTx (36 Gy)
Surgery, 3DCRTx (60,4 Gy), CTx (Docetaxel/
Doxorubicin/Cyclophosphamide)
ST-RTx (90 Gy)
Died 2018
♀
ST-RTx (20 Gy)
Surgery, WBRTx (30 Gy)
Age: 45 years
Diagnosis of Brain Mts 2014
Primary: Melanoma 2009
Diagnosis of Mts other than Brain 2013
Age: 67 years
Age: 71 years
CTx (Pembrolizumab)
Surgery, CTx (Interferon)
Surgery
♂
Lost to follow up 2020
ST-RTx (60 Gy)
Age: 73 years
Diagnosis of Brain Mts 2015
Primary: Melanoma 2012
Primary: Melanoma 2012
Diagnosis of Mts other than Brain 2012
Diagnosis of Mts other than Brain 2012
Age: 58 years
Surgery, CTx (Interferon)
Lost to follow up 2018
♂
Surgery, 3DCRTx (36Gy)
Age: 60 years
Diagnosis of Brain Mts 2014
Primary: Lung Cancer 2013
Diagnosis of Mts other than Brain 2013
Age: 39 years
ST-RTx (30 Gy)
CTx (Cisplatin/
ST-RTx (20 Gy)
WBRTx (30 Gy)
Permetrexed)
Died 2017
♀
ST-RTx (56 Gy)
ST-RTx (20Gy)
ST-RTx (100 Gy)
ST-RTx (80 Gy)
Age: 39 years
Diagnosis of Brain Mts 2013
0
1
5
2
15
10
4
13
8
11
6
7
9
12
14
3
16
Years after diagnosis

## Slide 4
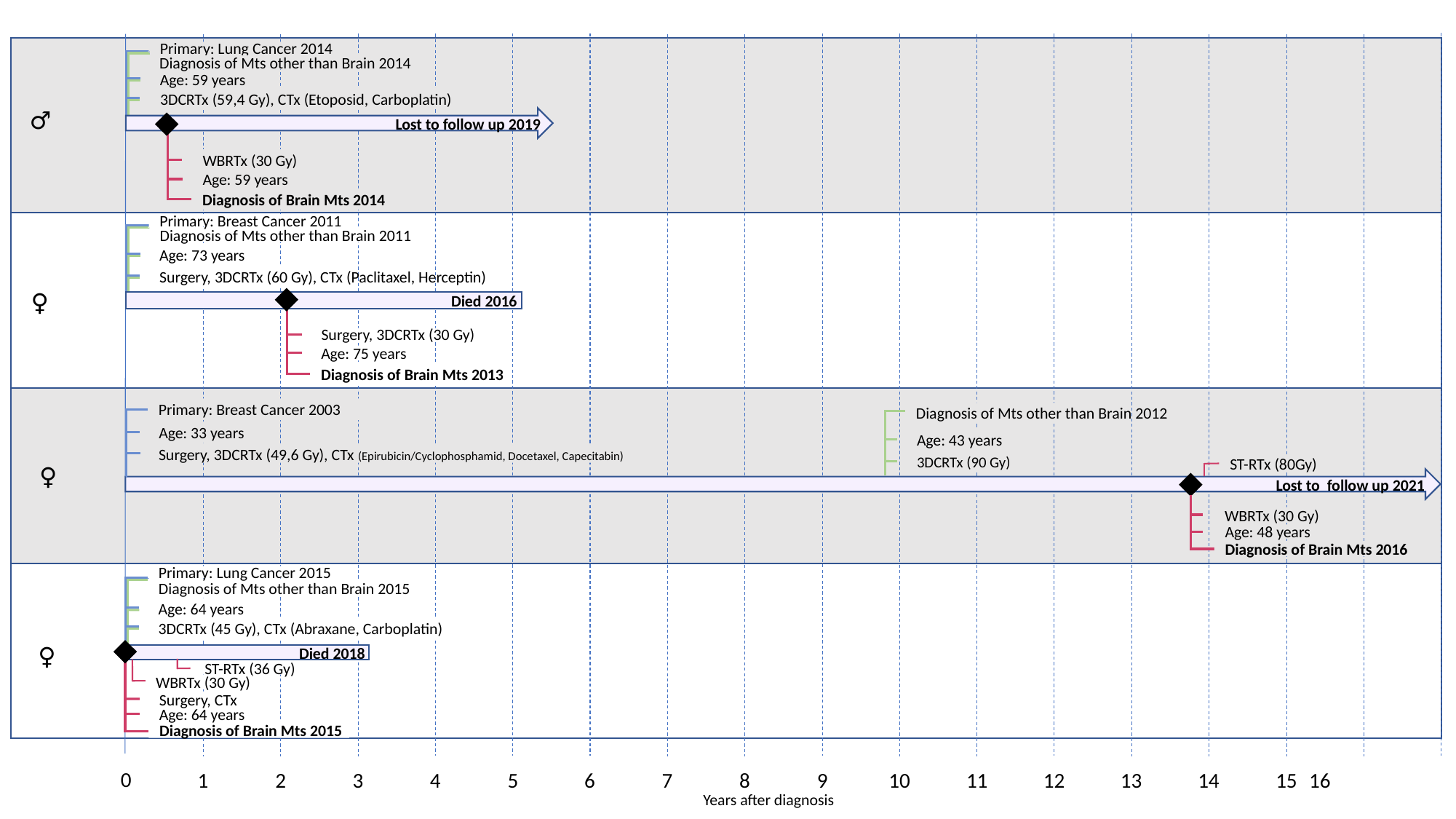

Primary: Lung Cancer 2014
Diagnosis of Mts other than Brain 2014
Age: 59 years
3DCRTx (59,4 Gy), CTx (Etoposid, Carboplatin)
♂
Lost to follow up 2019
WBRTx (30 Gy)
Age: 59 years
Diagnosis of Brain Mts 2014
Primary: Breast Cancer 2011
Diagnosis of Mts other than Brain 2011
Age: 73 years
Surgery, 3DCRTx (60 Gy), CTx (Paclitaxel, Herceptin)
Died 2016
♀
Surgery, 3DCRTx (30 Gy)
Age: 75 years
Diagnosis of Brain Mts 2013
Primary: Breast Cancer 2003
Diagnosis of Mts other than Brain 2012
Age: 33 years
Age: 43 years
Surgery, 3DCRTx (49,6 Gy), CTx (Epirubicin/Cyclophosphamid, Docetaxel, Capecitabin)
3DCRTx (90 Gy)
ST-RTx (80Gy)
♀
Lost to follow up 2021
WBRTx (30 Gy)
Age: 48 years
Diagnosis of Brain Mts 2016
Primary: Lung Cancer 2015
Diagnosis of Mts other than Brain 2015
Age: 64 years
3DCRTx (45 Gy), CTx (Abraxane, Carboplatin)
Died 2018
♀
 ST-RTx (36 Gy)
WBRTx (30 Gy)
Surgery, CTx
Age: 64 years
Diagnosis of Brain Mts 2015
0
1
5
2
15
10
4
13
8
11
6
7
9
12
14
3
16
Years after diagnosis

## Slide 5
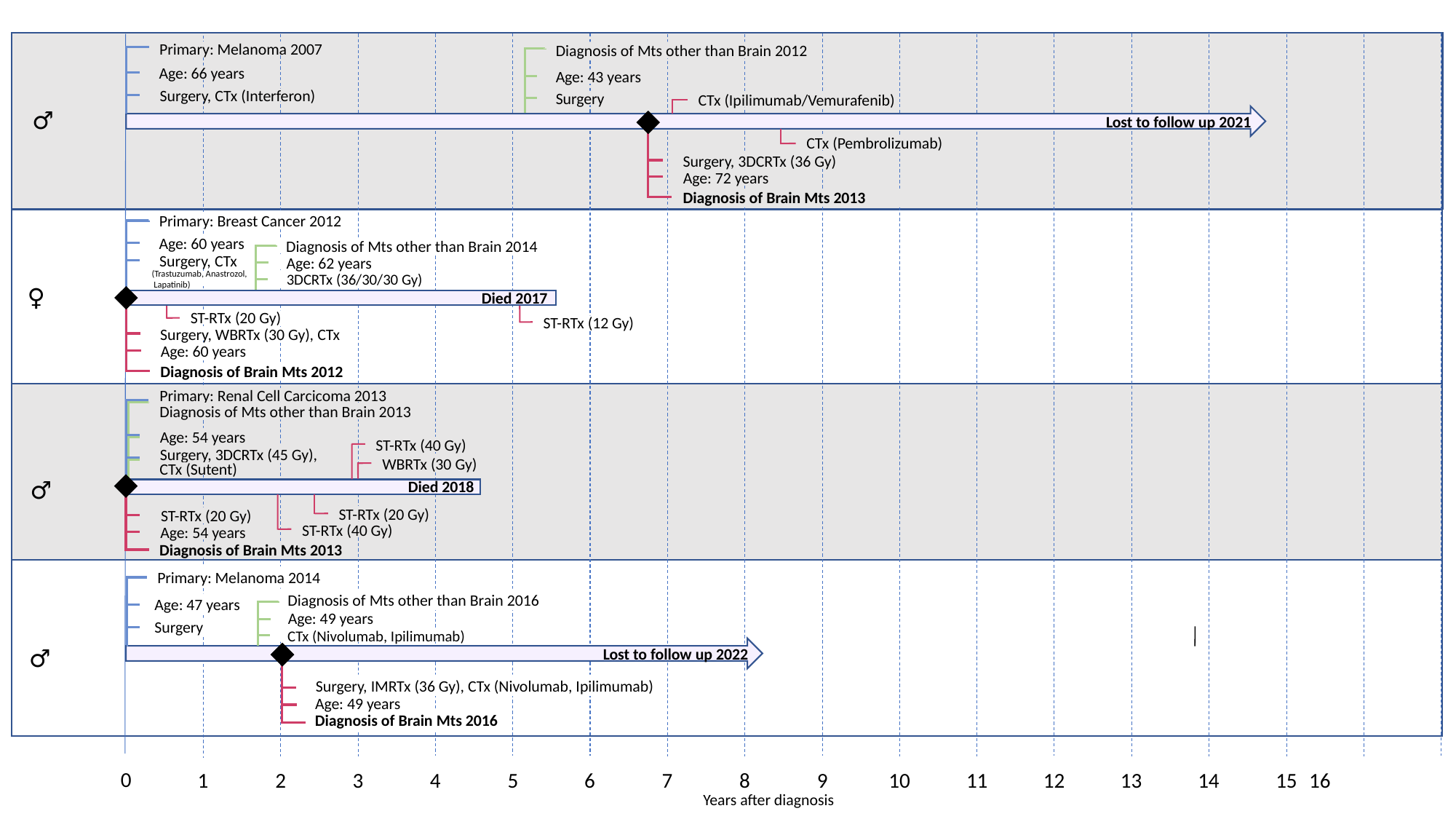

Primary: Melanoma 2007
Diagnosis of Mts other than Brain 2012
Age: 66 years
Age: 43 years
Surgery, CTx (Interferon)
Surgery
CTx (Ipilimumab/Vemurafenib)
♂
Lost to follow up 2021
CTx (Pembrolizumab)
Surgery, 3DCRTx (36 Gy)
Age: 72 years
Diagnosis of Brain Mts 2013
Primary: Breast Cancer 2012
Age: 60 years
Diagnosis of Mts other than Brain 2014
Surgery, CTx
Age: 62 years
(Trastuzumab, Anastrozol,
 Lapatinib)
3DCRTx (36/30/30 Gy)
♀
Died 2017
ST-RTx (20 Gy)
ST-RTx (12 Gy)
Surgery, WBRTx (30 Gy), CTx
Age: 60 years
Diagnosis of Brain Mts 2012
Primary: Renal Cell Carcicoma 2013
Diagnosis of Mts other than Brain 2013
Age: 54 years
ST-RTx (40 Gy)
Surgery, 3DCRTx (45 Gy),
WBRTx (30 Gy)
CTx (Sutent)
Died 2018
♂
ST-RTx (20 Gy)
ST-RTx (20 Gy)
ST-RTx (40 Gy)
Age: 54 years
Diagnosis of Brain Mts 2013
Primary: Melanoma 2014
Diagnosis of Mts other than Brain 2016
Age: 47 years
Age: 49 years
Surgery
CTx (Nivolumab, Ipilimumab)
Lost to follow up 2022
♂
Surgery, IMRTx (36 Gy), CTx (Nivolumab, Ipilimumab)
Age: 49 years
Diagnosis of Brain Mts 2016
0
1
5
2
15
10
4
13
8
11
6
7
9
12
14
3
16
Years after diagnosis

## Slide 6
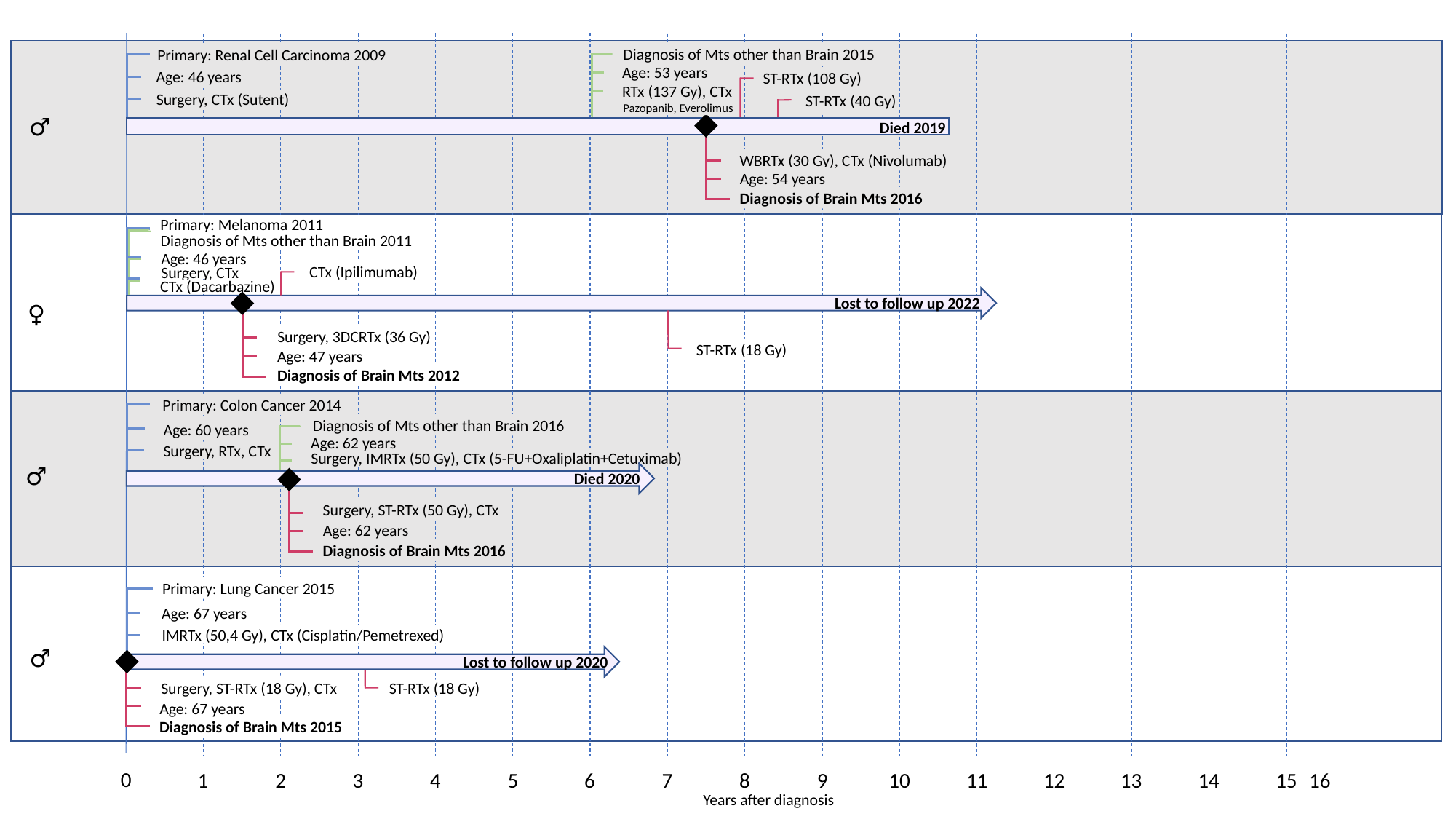

Diagnosis of Mts other than Brain 2015
Primary: Renal Cell Carcinoma 2009
Age: 53 years
Age: 46 years
ST-RTx (108 Gy)
RTx (137 Gy), CTx
ST-RTx (40 Gy)
Surgery, CTx (Sutent)
Pazopanib, Everolimus
Died 2019
♂
WBRTx (30 Gy), CTx (Nivolumab)
Age: 54 years
Diagnosis of Brain Mts 2016
Primary: Melanoma 2011
Diagnosis of Mts other than Brain 2011
Age: 46 years
CTx (Ipilimumab)
Surgery, CTx
CTx (Dacarbazine)
Lost to follow up 2022
♀
Surgery, 3DCRTx (36 Gy)
ST-RTx (18 Gy)
Age: 47 years
Diagnosis of Brain Mts 2012
Primary: Colon Cancer 2014
Diagnosis of Mts other than Brain 2016
Age: 60 years
Age: 62 years
Surgery, RTx, CTx
Surgery, IMRTx (50 Gy), CTx (5-FU+Oxaliplatin+Cetuximab)
Died 2020
♂
Surgery, ST-RTx (50 Gy), CTx
Age: 62 years
Diagnosis of Brain Mts 2016
Primary: Lung Cancer 2015
Age: 67 years
IMRTx (50,4 Gy), CTx (Cisplatin/Pemetrexed)
♂
Lost to follow up 2020
Surgery, ST-RTx (18 Gy), CTx
ST-RTx (18 Gy)
Age: 67 years
Diagnosis of Brain Mts 2015
0
1
5
2
15
10
4
13
8
11
6
7
9
12
14
3
16
Years after diagnosis

## Slide 7
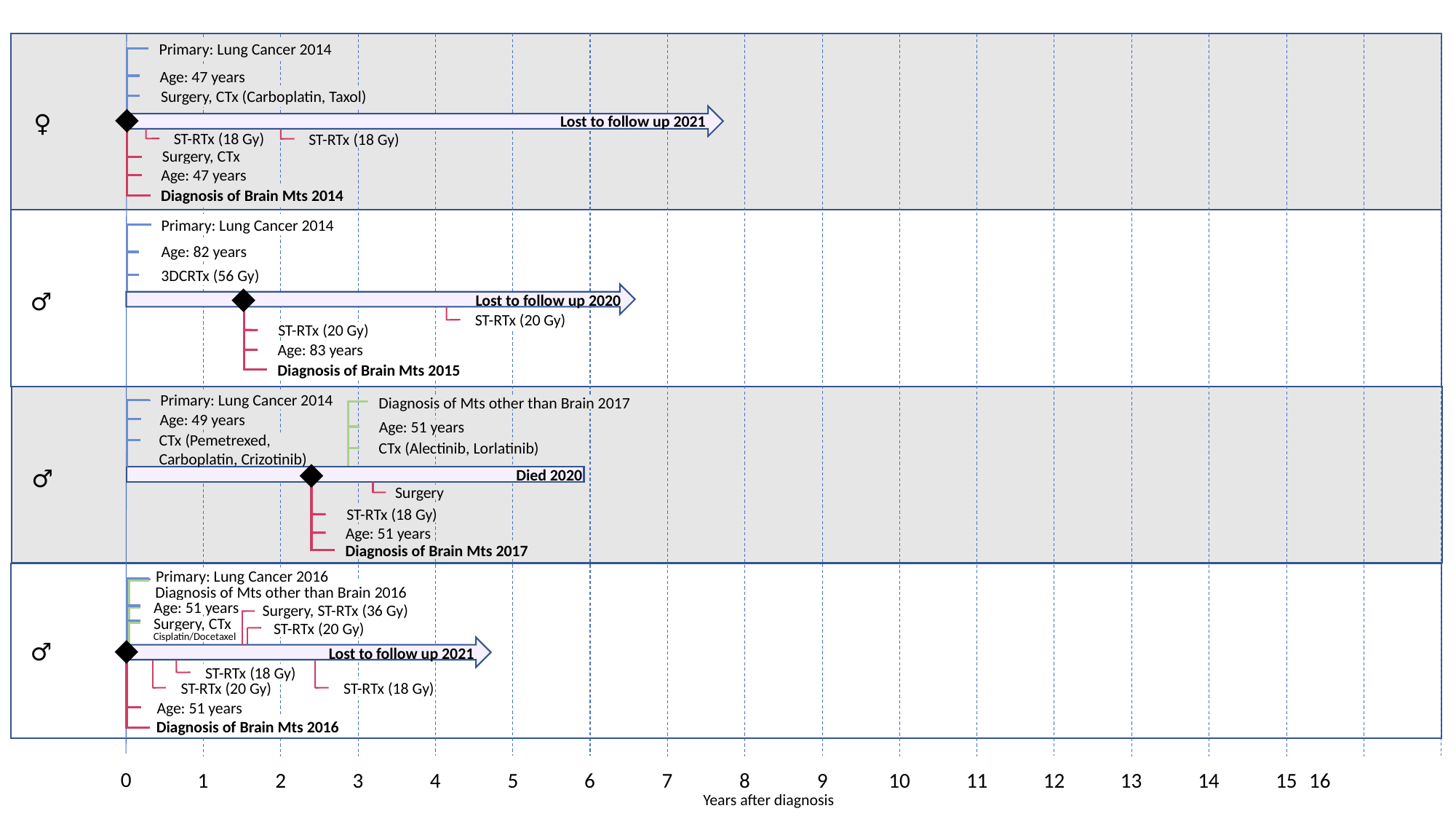

Primary: Lung Cancer 2014
Age: 47 years
Surgery, CTx (Carboplatin, Taxol)
Lost to follow up 2021
♀
 ST-RTx (18 Gy)
 ST-RTx (18 Gy)
 Surgery, CTx
Age: 47 years
Diagnosis of Brain Mts 2014
Primary: Lung Cancer 2014
Age: 82 years
3DCRTx (56 Gy)
Lost to follow up 2020
♂
ST-RTx (20 Gy)
ST-RTx (20 Gy)
Age: 83 years
Diagnosis of Brain Mts 2015
Primary: Lung Cancer 2014
Diagnosis of Mts other than Brain 2017
Age: 49 years
Age: 51 years
CTx (Pemetrexed,
Carboplatin, Crizotinib)
CTx (Alectinib, Lorlatinib)
Died 2020
♂
Surgery
ST-RTx (18 Gy)
Age: 51 years
Diagnosis of Brain Mts 2017
Primary: Lung Cancer 2016
Diagnosis of Mts other than Brain 2016
Age: 51 years
Surgery, ST-RTx (36 Gy)
Surgery, CTx
ST-RTx (20 Gy)
Cisplatin/Docetaxel
♂
Lost to follow up 2021
ST-RTx (18 Gy)
ST-RTx (18 Gy)
ST-RTx (20 Gy)
Age: 51 years
Diagnosis of Brain Mts 2016
0
1
5
2
15
10
4
13
8
11
6
7
9
12
14
3
16
Years after diagnosis

## Slide 8
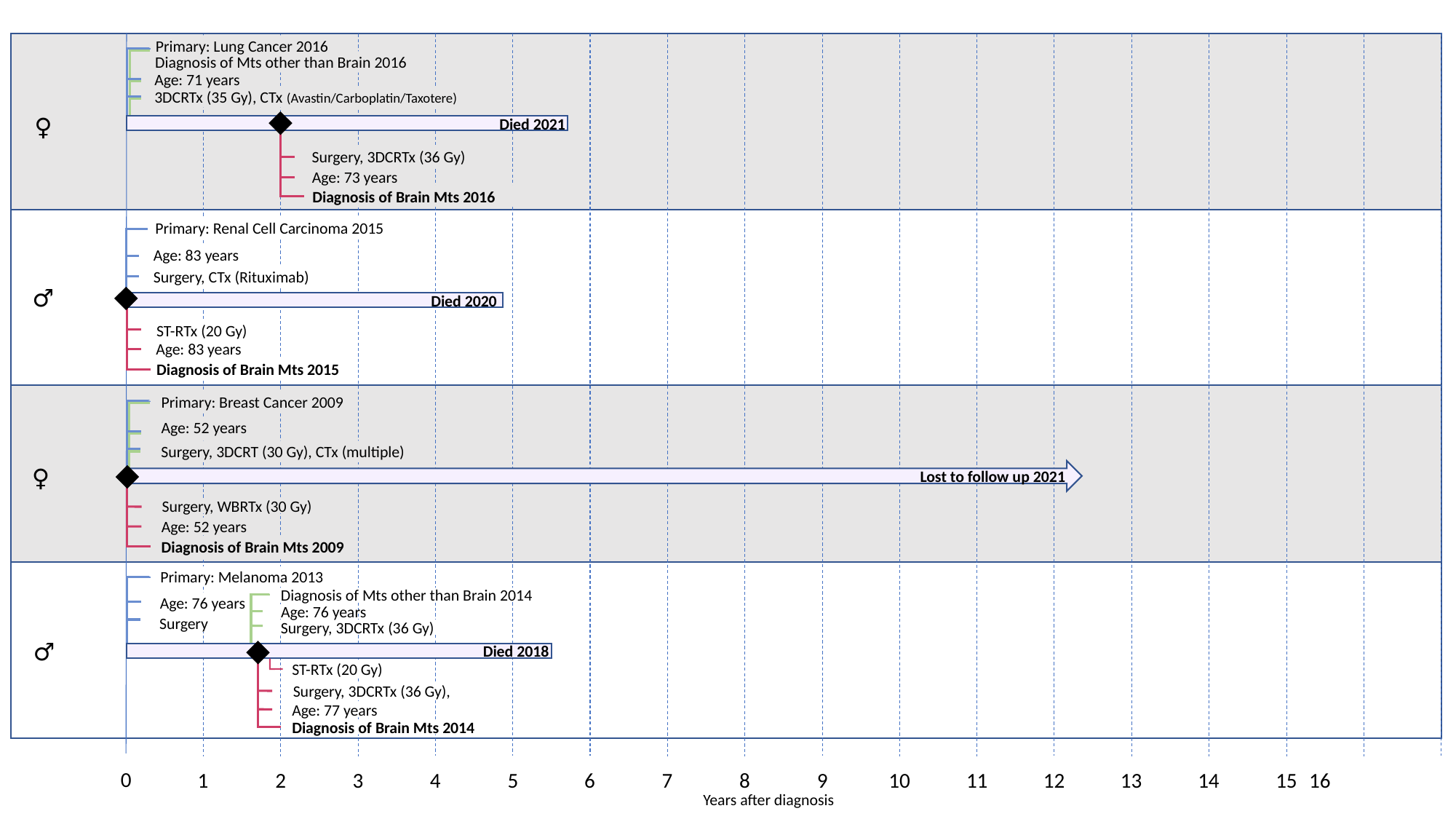

Primary: Lung Cancer 2016
Diagnosis of Mts other than Brain 2016
Age: 71 years
3DCRTx (35 Gy), CTx (Avastin/Carboplatin/Taxotere)
Died 2021
♀
Surgery, 3DCRTx (36 Gy)
Age: 73 years
Diagnosis of Brain Mts 2016
Primary: Renal Cell Carcinoma 2015
Age: 83 years
Surgery, CTx (Rituximab)
♂
Died 2020
ST-RTx (20 Gy)
Age: 83 years
Diagnosis of Brain Mts 2015
Primary: Breast Cancer 2009
Age: 52 years
Surgery, 3DCRT (30 Gy), CTx (multiple)
Lost to follow up 2021
♀
Surgery, WBRTx (30 Gy)
Age: 52 years
Diagnosis of Brain Mts 2009
Primary: Melanoma 2013
Diagnosis of Mts other than Brain 2014
Age: 76 years
Age: 76 years
Surgery
Surgery, 3DCRTx (36 Gy)
Died 2018
♂
ST-RTx (20 Gy)
Surgery, 3DCRTx (36 Gy),
Age: 77 years
Diagnosis of Brain Mts 2014
0
1
5
2
15
10
4
13
8
11
6
7
9
12
14
3
16
Years after diagnosis

## Slide 9
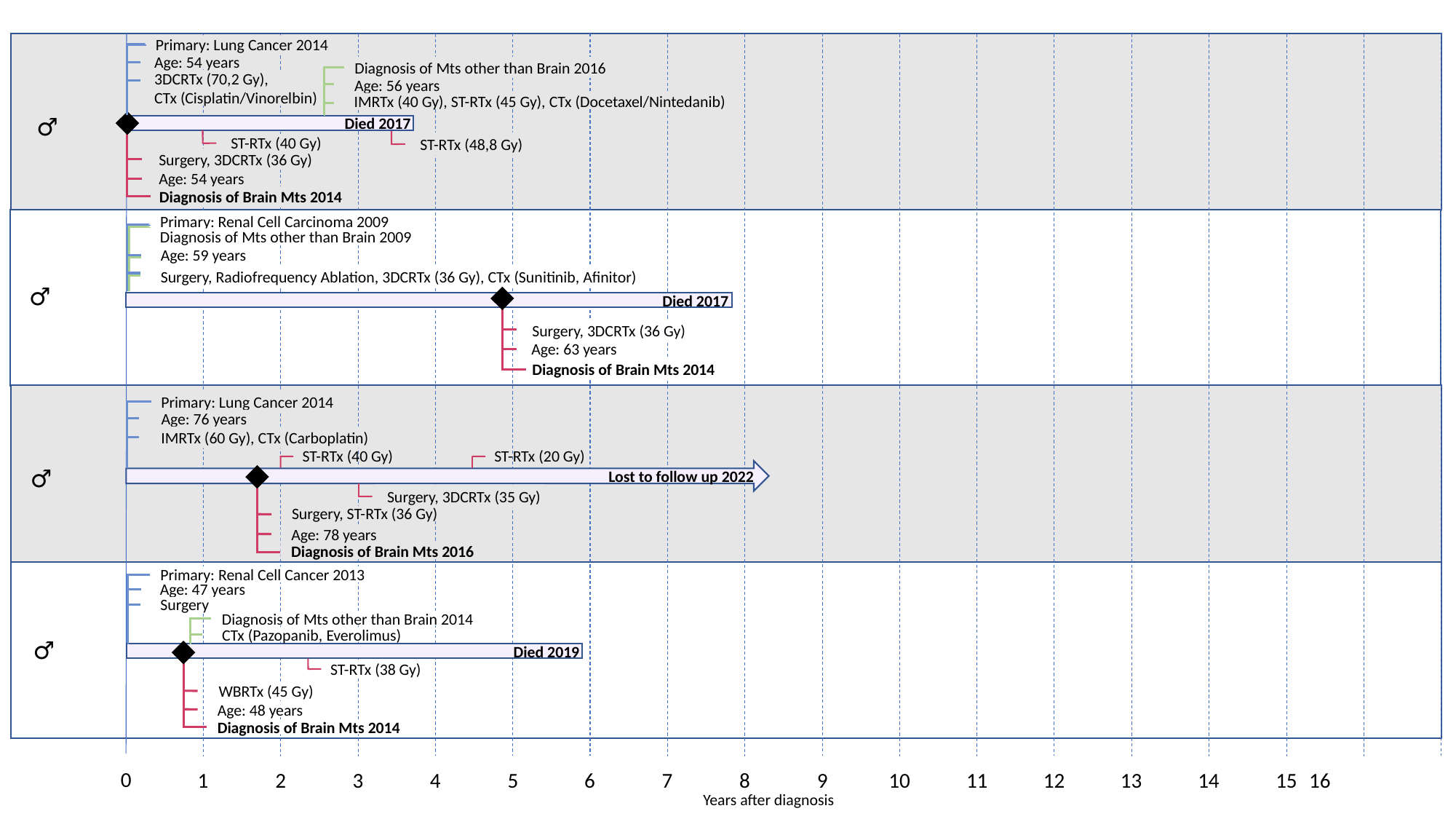

Primary: Lung Cancer 2014
#
Age: 54 years
Diagnosis of Mts other than Brain 2016
3DCRTx (70,2 Gy),
CTx (Cisplatin/Vinorelbin)
Age: 56 years
IMRTx (40 Gy), ST-RTx (45 Gy), CTx (Docetaxel/Nintedanib)
♂
Died 2017
ST-RTx (48,8 Gy)
ST-RTx (40 Gy)
Surgery, 3DCRTx (36 Gy)
Age: 54 years
Diagnosis of Brain Mts 2014
Primary: Renal Cell Carcinoma 2009
Diagnosis of Mts other than Brain 2009
Age: 59 years
Surgery, Radiofrequency Ablation, 3DCRTx (36 Gy), CTx (Sunitinib, Afinitor)
♂
Died 2017
Surgery, 3DCRTx (36 Gy)
Age: 63 years
Diagnosis of Brain Mts 2014
Primary: Lung Cancer 2014
Age: 76 years
IMRTx (60 Gy), CTx (Carboplatin)
ST-RTx (20 Gy)
ST-RTx (40 Gy)
Lost to follow up 2022
♂
Surgery, 3DCRTx (35 Gy)
Surgery, ST-RTx (36 Gy)
Age: 78 years
Diagnosis of Brain Mts 2016
Primary: Renal Cell Cancer 2013
Age: 47 years
Surgery
Diagnosis of Mts other than Brain 2014
CTx (Pazopanib, Everolimus)
♂
Died 2019
ST-RTx (38 Gy)
WBRTx (45 Gy)
Age: 48 years
Diagnosis of Brain Mts 2014
0
1
5
2
15
10
4
13
8
11
6
7
9
12
14
3
16
Years after diagnosis

## Slide 10
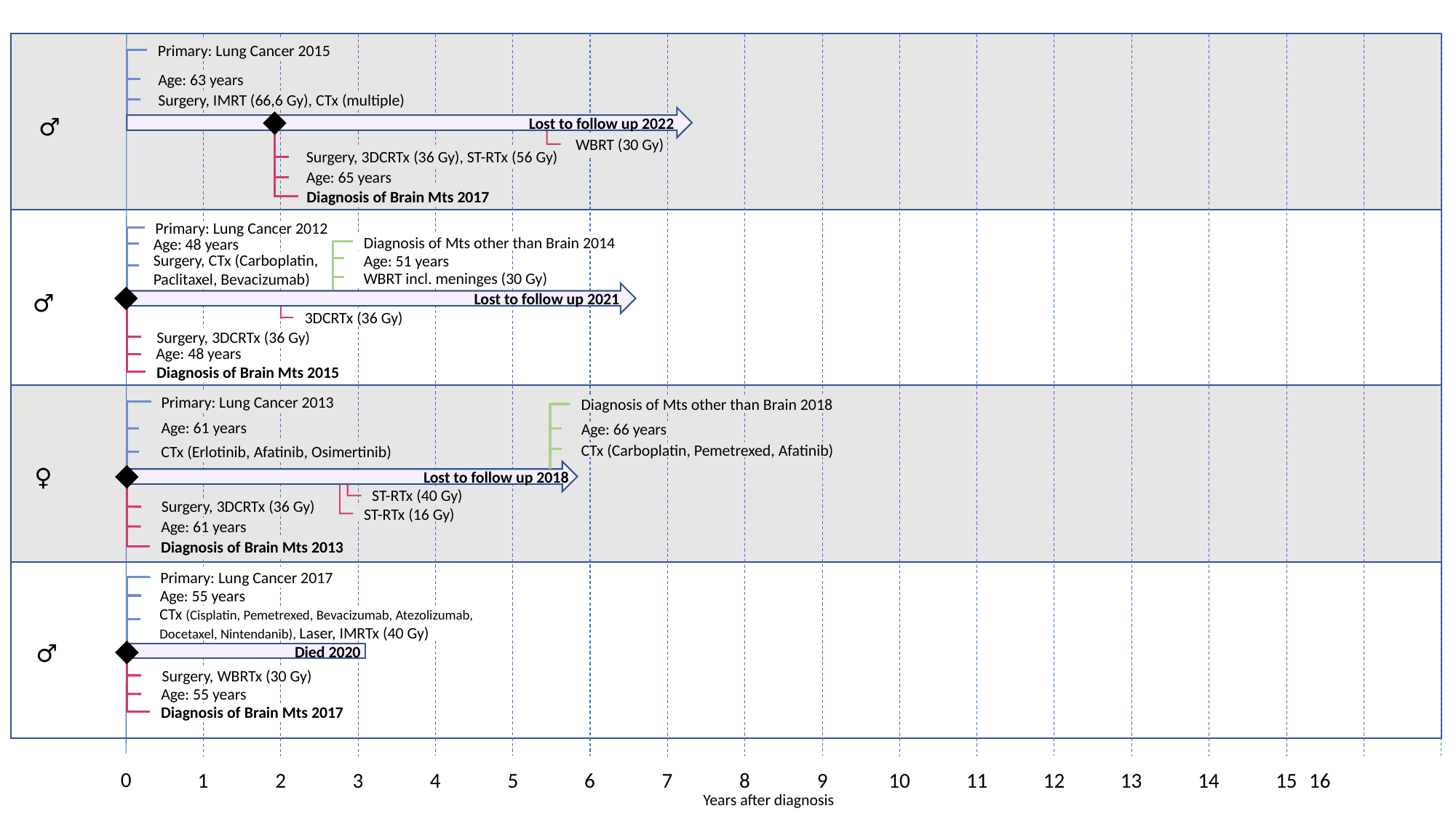

Primary: Lung Cancer 2015
#
Age: 63 years
Surgery, IMRT (66,6 Gy), CTx (multiple)
Lost to follow up 2022
♂
WBRT (30 Gy)
Surgery, 3DCRTx (36 Gy), ST-RTx (56 Gy)
Age: 65 years
Diagnosis of Brain Mts 2017
Primary: Lung Cancer 2012
Diagnosis of Mts other than Brain 2014
Age: 48 years
Age: 51 years
Surgery, CTx (Carboplatin,
Paclitaxel, Bevacizumab)
WBRT incl. meninges (30 Gy)
Lost to follow up 2021
♂
3DCRTx (36 Gy)
Surgery, 3DCRTx (36 Gy)
Age: 48 years
Diagnosis of Brain Mts 2015
Primary: Lung Cancer 2013
Diagnosis of Mts other than Brain 2018
Age: 61 years
Age: 66 years
CTx (Erlotinib, Afatinib, Osimertinib)
CTx (Carboplatin, Pemetrexed, Afatinib)
♀
Lost to follow up 2018
ST-RTx (40 Gy)
Surgery, 3DCRTx (36 Gy)
ST-RTx (16 Gy)
Age: 61 years
Diagnosis of Brain Mts 2013
Primary: Lung Cancer 2017
Age: 55 years
CTx (Cisplatin, Pemetrexed, Bevacizumab, Atezolizumab,
Docetaxel, Nintendanib), Laser, IMRTx (40 Gy)
Died 2020
♂
Surgery, WBRTx (30 Gy)
Age: 55 years
Diagnosis of Brain Mts 2017
0
1
5
2
15
10
4
13
8
11
6
7
9
12
14
3
16
Years after diagnosis

## Slide 11
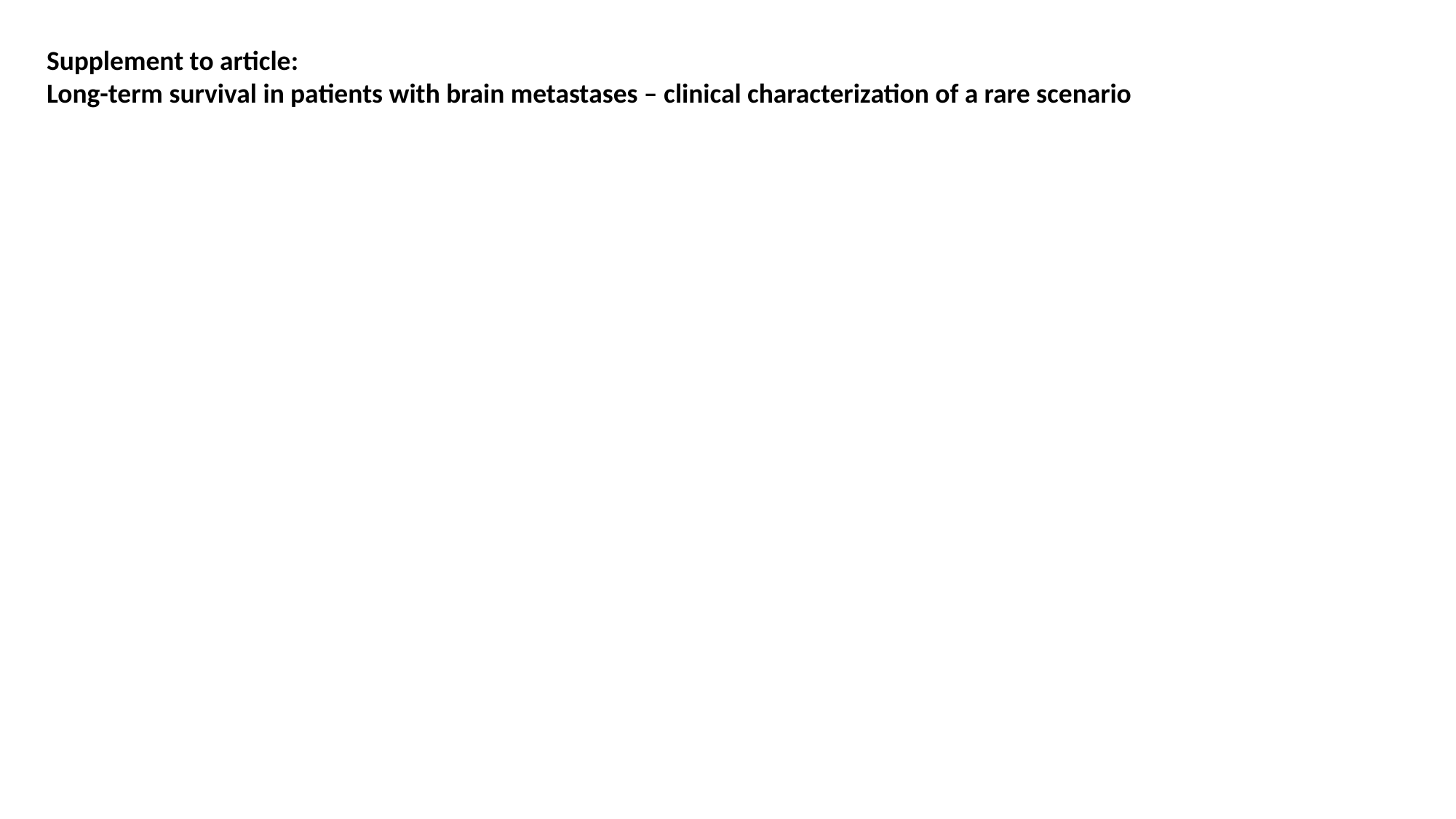

Supplement to article:
Long-term survival in patients with brain metastases – clinical characterization of a rare scenario
